# Neuroproteomics in Epilepsy: What Do We Know so Far?

**DOI:** 10.3389/fnmol.2020.604158

**Published:** 2021-01-07

**Authors:** Amanda M. do Canto, Amanda Donatti, Jaqueline C. Geraldis, Alexandre B. Godoi, Douglas C. da Rosa, Iscia Lopes-Cendes

**Affiliations:** ^1^Department of Medical Genetics and Genomic Medicine, School of Medical Sciences, University of Campinas (UNICAMP), Campinas, Brazil; ^2^Brazilian Institute of Neuroscience and Neurotechnology (BRAINN), Campinas, Brazil

**Keywords:** mesial temporal lobe epilepsy, hippocampal sclerosis, malformations of cortical development, proteomics, epileptogenesis, seizures

## Abstract

Epilepsies are chronic neurological diseases that affect approximately 2% of the world population. In addition to being one of the most frequent neurological disorders, treatment for patients with epilepsy remains a challenge, because a proportion of patients do not respond to the antiseizure medications that are currently available. This results in a severe economic and social burden for patients, families, and the healthcare system. A characteristic common to all forms of epilepsy is the occurrence of epileptic seizures that are caused by abnormal neuronal discharges, leading to a clinical manifestation that is dependent on the affected brain region. It is generally accepted that an imbalance between neuronal excitation and inhibition generates the synchronic electrical activity leading to seizures. However, it is still unclear how a normal neural circuit becomes susceptible to the generation of seizures or how epileptogenesis is induced. Herein, we review the results of recent proteomic studies applied to investigate the underlying mechanisms leading to epilepsies and how these findings may impact research and treatment for these disorders.

## Introduction

Epilepsies are a group of life-threatening chronic neurological diseases affecting over 50 million people worldwide (Hauser et al., 1996; Borges et al., 2004; World Federation of Neurology and World Health Organization, 2017). While heterogeneous, these disorders have in common the presence of spontaneous recurrent seizures (Laidlaw, 1988; England et al., 2012). Epileptic seizures are induced by abnormal neuronal discharges, which lead to variable clinical manifestations that depend on the affected brain regions (Fisher et al., 2014; Devinsky et al., 2018). Despite the high number of antiseizure drugs (ASDs) available, some patients do not respond to treatment and have so-called pharmacoresistant or refractory epilepsy (Kwan et al., 2010). Also, the current available ASDs target only the symptoms rather than the cause of epilepsy, thus requiring the continuous use of ASDs, which can affect overall brain functioning and lead to many side effects (Kwan et al., 2010; Löscher and Schmidt, 2011; Brodie et al., 2012; Crepeau and Sirven, 2017). Therefore, understanding the biological mechanisms underlying human epilepsy is critical to developing new and more effective medications to treat these patients.

Because of its complexity, different mechanisms can be involved in the various types of epilepsy, such as the imbalance between inhibitory and excitatory neurotransmission, high levels of inflammation, neuronal damage in specific brain regions, and abnormalities in neuronal and cortical development (Crespel et al., 2002; Blümcke et al., 2013; Devinsky et al., 2013, 2018; Staley, 2015; Gales and Prayson, 2017; Ye and Kaszuba, 2017). All these mechanisms may be caused by environmental or genetic factors and, most likely, by interactions among them. Genetic and developmental abnormalities are more prevalent in children, whereas, in adults and elderly patients, acquired causes, such as brain tumors, trauma, and stroke, are more frequent (Allone et al., 2017; Magalhães et al., 2019; Thijs et al., 2019). However, it is still unclear how a normal neural circuit becomes more susceptible to the generation of seizures or how epileptogenesis is induced. Also, it is unlikely that a single factor or mechanism can lead to all the changes seen in the various types of epilepsy.

**Figure 1:**
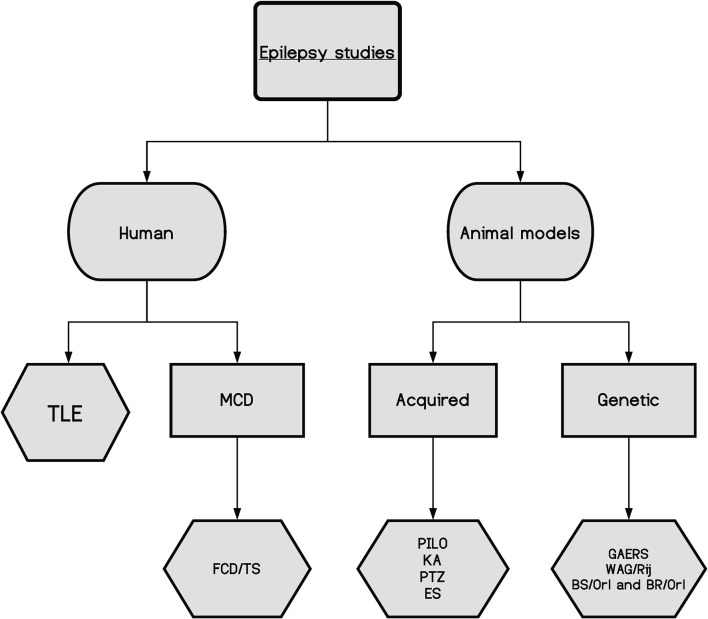
A schematic of the main epilepsy studies and their divisions, highlighting the complexity of the disease. TLE, temporal lobe epilepsy; MCD, malformations of cortical development; FCD, focal cortical dysplasia; TS, tuberous sclerosis; PILO, pilocarpine model; KA, kainic acid model; PTZ, pentylenetetrazol model; ES, electrical stimulation models; GAERS, genetic absence epilepsy rats from Strasbourg; WAG/Rjj, Wistar Albino Glaxo/Rijswijk rat; BS/Orl and BR/Orl, mouse strains.

Even with all the significant developments achieved over the last few decades in biomedical sciences, there is still a substantial part of the cellular machinery that remains poorly understood. Therefore, by studying proteins and their dynamics, presence, and abundance, one can gain new insights into cellular functions in normal and pathological tissues. Hence, proteomic studies can help researchers understand the biological mechanisms leading to epilepsy, the development of new treatments, and the discovery of biomarkers of disease, which may assist in predicting the best responses to treatment. Proteomics is best performed in the target tissue where the molecular changes may take place; thus, in epilepsy, there are two main sources: human tissue, primarily resected brain samples obtained from epilepsy surgery, and tissue from animal models of epilepsy, which can be induced by different strategies or genetic models (Figure 1; Grone and Baraban, 2015; Becker, 2018; Devinsky et al., 2018).

**Figure 2:**
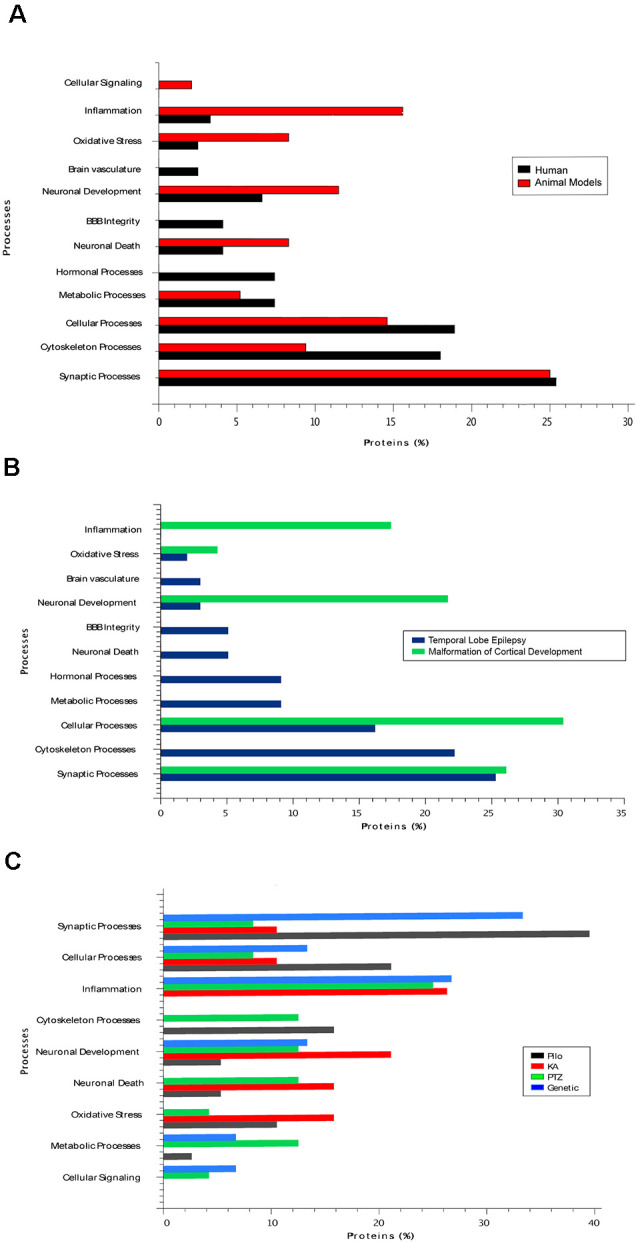
**(A)** The main biological processes associated with epilepsy in human tissue and in tissues from rodent models of the disease. **(B)** The biological processes present in two types of human epilepsy, namely temporal lobe epilepsy (TLE) and malformations of cortical development. **(C)** The main biological processes present in studies with different animal models of epilepsy. Pilo, pilocarpine model; KA, kainic acid model; PTZ, pentylenetetrazol model.

Herein, we reviewed the recent findings of proteomic studies on epilepsy and discussed how these findings might impact research and the clinical management of epileptic patients. A summary of the main neuroproteomics findings in epilepsy is presented in Table 1 and Figure 2. The studies are reviewed in chronological order because there has been a significant impact from technological developments in proteomics over the past few years. Please note that the proteins discussed in the text are presented in their abbreviated form. Table 1 provides the full name of the discussed proteins.

**Table 1:** The main proteins associated with epilepsy and their contributions to the phenotype.

Tissue	Method	Protein	ID	Relation	Biological effect	Reference
**Focal epilepsies**						
*Temporal lobe epilepsy*						
Hipp	SPCT	Mitochondrial complex I (NADH dehydrogenase).	NADH	Decrease	Excitability and selective neuronal vulnerability.	Kunz et al. (2000)
					Increased calcium ions in the cytosolic environment.	
Hipp	IHC + WB	Neuronal nitric oxide synthase.	nNOS	Decrease	Decreased neurotransmitter release and ion channel dynamics.	Leite et al. (2002)
Hipp	2DE + MALDI/TOF	Collapsin response mediator protein 2.	CRMP-2	Decrease	Aberrant axonal reorganization.	Czech et al. (2004)
Hipp	2DE + MS	Acyl-CoA thioester hydrolase.	ACOT	Decrease	Fatty acid metabolism.	Yang et al. (2004)
Hipp	2DE + MALDI-TOF	Apolipoprotein A-I	ApoAI	Increase	BBB integrity	Yang et al. (2005) and He et al. (2006)
Hipp	2DE + MALDI-TOF	Dimethylarginine dimethylaminohydrolase 1.	DDAH1	Decrease	Decrease of neurotransmitter release.	He et al. (2006)
Hipp	2DE + MALDI-TOF	Glutamine synthetase	GS	Decrease	Excitotoxicity	He et al. (2006)
Hipp	2DE + MALDI-TOF	4-aminobutyrate transaminase.	ABAT	Decrease	Glutamate metabolism	He et al. (2006)
Hipp	2DE + MALDI-TOF	Uracil DNA glycosylase	UNG	Decrease	Neuronal death	He et al. (2006)
Hipp	2DE + MALDI-TOF	Silent information regulator 2 protein.	SIR2	Decrease	DNA repair and cytoprotection.	He et al. (2006)
Hipp	2DE + MALDI-TOF	Alpha-crystallin B	CRYAB	Decrease	Inhibits the aggregation of unfolded proteins.	He et al. (2006) and Keren-Aviram et al. (2018)
Hipp	2DE + MALDI-TOF	Neurofilament 3/Septin-8/alpha tubulin, isoform 1/beta tubulin.	NEF3/Sept8/α-1-tubulin/β-tubulin.	Decrease	Cytoskeleton damage	He et al. (2006)
Hipp	2DE + MALDI-TOF	Dihydropyrimidinase-like 2	DPYSL2	Decrease	Cytoskeleton damage	He et al. (2006) and Persike et al. (2012)
Hipp	2DE + MALDI-TOF	Beta tubulin, isoform 5/Ezrin/Vinculin	β5-tubulin/EZR/VCL.	Increase	Cytoskeleton damage	Yang et al. (2006)
Hipp	2DE + MALDI-TOF	Profilin II	PFN2	Decrease	Cytoskeleton damage	Yang et al. (2006)
Hipp	2DE + MALDI-TOF	Neuronal tropomodulin	TMOD2	Decrease	Neuronal death and cytoskeleton integrity.	Yang et al. (2006)
Hipp	2DE + MALDI-TOF	Peroxiredoxin 3/ Peroxiredoxin 6	PRDX3/PRDX6	Decrease/increase	Attack to reactive oxygen species.	Yang et al. (2006)
Hipp	2DE + MALDI-TOF	T complex protein I alpha	TCP1	Decrease	Cytoskeleton structure	Yang et al. (2006)
Hipp	2DE + MALDI-TOF	Mitogen-activated protein kinase kinase 1.	MPKK1	Decrease	Phosphorylation of cytoskeleton proteins.	Yang et al. (2006)
Hipp	2DE + MALDI-TOF	Synaptotagmin I	Syt I	Decrease	Regulation of neurotransmitter release and transport.	Yang et al. (2006)
Hipp	2DE + MALDI-TOF	Alpha-synuclein	SNCA	Decrease/increase	Regulation of neurotransmitter release and transport.	Yang et al. (2006) and Keren-Aviram et al. (2018)
Hipp	2DE + MALDI-TOF	Spectrin alpha chain, isoform 3	SPTAN1	Decrease	Electrophysiological activity of the NMDA receptor.	Persike et al. (2012)
Hipp	2DE + MALDI-TOF	Dihydrolipoyllysine-residue acetyltransferase component of pyruvate dehydrogenase complex, mitochondrial.	DLAT	Increase	Metabolic disturbance and oxidative damage.	Persike et al. (2012)
Hipp	2DE + MALDI-TOF	Parkinson disease protein 7	PARK7	Presence	Protection against oxidative stress and cell death in this condition.	Persike et al. (2012)
Hipp	2DE + MALDI-TOF	Glutathione S transferase P	GSTP1	Presence	Hydrophobic electrophiles maintenance.	Persike et al. (2012)
Hipp	2DE + MALDI-TOF	Heat shock-related 70 kDa protein 2	HSPA2	Increase	Cytoskeleton development.	Persike et al. (2012)
Hipp	MALDI MSI/MS	Neuropeptide Y	NPY	Increase	Seizure induces neurogenesis.	Mériaux et al. (2014)
Hipp	MALDI MSI/MS	Somatostatin 1	Somatostatin	Increase	Neuromodulator	Mériaux et al. (2014)
Hipp	LC-LTQ MS/MS	Leucine-rich glioma inactivated 1.	LIGI1	Presence	Male autosomal dominant partial epilepsy with auditory features (ADPEAF).	Mériaux et al. (2014)
Neocort	LC/MS	Collapsin response mediator protein 2.	CRMP-2	Increase	Synaptic plasticity related to the epileptic neocortex.	Keren-Aviram et al. (2018)
Neocort	LC/MS	Glial fibrillary acidic protein.	GFAP	Decrease	BBB function and cell communication.	Keren-Aviram et al. (2018)
Neocort	LC/MS	Guanine nucleotide-binding protein G(o) subunit α/LIM and SH3 protein 1.	GNAO1/LASP1	Decrease	Astrocyte origin	Keren-Aviram et al. (2018)
Neocort	LC/MS	Synapsin II/Stathmin 1	SYN2/STMN1	Increase	Neuritogenesis and synaptic transmission.	Keren-Aviram et al. (2018)
Hipp	LC/MS	O-GlcNAcase	GOA	Absence	Increase OGlcNacetylation in tissues.	Sánchez et al. (2019)
Hipp	IHC	Mitochondrial ATP synthase	ATP5B	Decrease	Neuronal cell maintenance	Mota et al. (2019)
*Focal cortical dysplasia*
Neocort	LC-MS/MS + WB + IHC	Fascin/dihydropyrimidinase-related protein 1/microtubule-associated protein 4.	FSCN1/CRMP1/MAP4	Increase	Neuron migration, neurite outgrowth, and abnormal dendrite orientation.	Qin et al. (2017)
Neocort	LC-MS/MS + WB + IHC	Protein NDRG1	NDRG1	Increase	Reactive oligodendroglial hyperplasia.	Qin et al. (2017)
Neocort	LC-MS/MS + WB + IHC	Peroxiredoxin-6	PRDX6	Decrease	Oxidative stress	Qin et al. (2017)
Neocort	LC-MS/MS + WB + IHC	Prosaposin	PSAP	Decrease	Neuroprotection/neurotoxicity.	Qin et al. (2017)
*Tuberous sclerosis*
Tubers	LC-MS/MS	Hamartin	TSC1	Decrease	Synaptic signaling and inflammation.	Dombkowski et al. (2016)
Neocort	LC-MS/MS	Brefeldin A-inhibited guanine nucleotide-exchange protein 2/neuronal migration protein doublecortin.	ARFGEF2/DCX	Decrease/increase	Intellectual disability and abnormal neuronal migration.	Liu et al. (2020)
Neocort	LC-MS/MS	Ubiquitin-like protein ATG12.	ATG12	Decrease	Autophagy	Liu et al. (2020)
Neocort	LC-MS/MS	Potassium voltage-gated channel subfamily A member 2.	KCNA2/KCNA2B	Decrease	Membrane excitability.	Liu et al. (2020)

*2DE, two-dimensional electrophoresis; BBB, blood-brain barrier; Hipp, hippocampus; IHC, immunohistochemistry; LC, liquid chromatography; MALDI, matrix-assisted laser desorption ionization; MS, mass spectrometry; Neocort, neocortex; SPCT, spectrophotometry; TOF, time of flight; WB, Western Blot*.

## Proteomic Studies in Human Epilepsy

In most proteomic studies, evaluating the affected tissue is an essential approach to measure molecular dynamics at a determined point in the time course of the disease (Al Diffalha et al., 2019). Thus, the availability of tissue to be studied by different proteomic techniques is a central issue in neuroproteomics. Human tissues are restricted in studies on epilepsy because brain tissue is only available *postmortem* or from surgery performed on patients with focal epilepsy who do not respond to treatment with ASDs. When using tissue from an autopsy, it is important to observe the *postmortem* interval (PMI), which is the time between death and the autopsy procedure. This time usually varies from 1 h to over 24 h. Given that autopsy material is widely used as control tissue in human neuroscience, tissue preservation must be considered and the PMI is a significant factor that can interfere with the tissue quality (Chandana et al., 2009; Blair et al., 2016). Some studies have investigated the impact of the PMI on biomolecule integrity and found that the *postmortem* protein changes are dependent on the evaluated protein. Even though studies suggest that proteins are stable in a short PMI (Nagy et al., 2015; Blair et al., 2016), comparison of *postmortem* and biopsy tissues requires caution because the identified molecular differences may be due to a confounding factor.

The two most important types of medically refractory focal epilepsy, which can benefit from surgical tissue resection, are temporal lobe epilepsy (TLE) and malformations of cortical development (Cendes, 2005; Kwan et al., 2010).

### Temporal Lobe Epilepsy

TLE is a common form of focal epilepsy in humans, with seizures originating in the temporal lobe (Cendes, 2005). In addition to seizures being frequent, a significant proportion of patients with TLE do not respond to treatment with ASDs, leading to the diagnosis of pharmacoresistant epilepsy in about 30% of these patients (Kwan et al., 2010; Allone et al., 2017; Luan et al., 2018). TLE can be divided into two sub-types: mesial temporal lobe epilepsy (MTLE), the most common and a usually severe form of epilepsy, and neocortical temporal lobe epilepsy (NTLE; Cendes, 2005; Tassi et al., 2009; Allone et al., 2017). In most patients with MTLE, seizure onset occurs in the hippocampus, entorhinal cortex, or amygdala (Cendes, 2005; Lévesque et al., 2018). Mesial temporal sclerosis (MTS), including hippocampal sclerosis (HS), is the main histopathological finding in these patients, and it is present in 60–70% of patients with MTLE who undergo a surgical procedure to treat medically refractory seizures. MTS is the histopathological term for neuronal loss and gliosis involving the hippocampus, and frequently also the amygdala, uncus, and parahippocampal gyrus (Blümcke et al., 1999, 2013; Cendes, 2005).

As described above, most proteomic studies using human tissue are derived from surgical specimens obtained from patients with MTLE who underwent resection of the mesial temporal structures to treat medically refractory epilepsy. It is known that dysregulation of synaptic processes is the most common mechanism in epileptic seizures. However, due to the disease’s complexity, several biological processes are probably interacting to determine the epileptogenic lesion seen in patients with MTLE, including energetic, signaling, and cytoskeleton pathways (Yang et al., 2004, 2006; Persike et al., 2012; Fukata and Fukata, 2017). The first studies to evaluate changes at the protein level related to epilepsy reported findings involved with abnormal neuronal excitability. In 2000, an enzymatic study analyzed, through spectrophotometry (SPCT), the levels of NADH:CoQ1 oxidoreductase and cytochrome *c* oxidase in hippocampal tissue of patients with MTLE (Kunz et al., 2000). There was a loss in complex I of the mitochondrial respiratory chain complex in the *Cornu Ammonis* CA(3) of the hippocampus and the dentate gyrus (DG). Alterations in complex I of this chain have been described as promoting changes in excitability and selective neuronal vulnerability in the hippocampal regions (Kunz et al., 2000; Yang et al., 2004). Kunz et al. (2000) analyzed 19 TLE samples and one control using histochemistry and electron microscopy. Despite having a good number of patient samples (*n* = 57), there was high heterogeneity among them. Some patients presented severe segmental loss in the hippocampus, others presented moderate hippocampal neuronal loss, and some even showed no signs of pathological abnormalities. Also, the authors analyzed only two controls, normal tissue obtained from a patient with a non-epileptogenic tumor within the mesial temporal lobe. Furthermore, the main focus of that study was enzymatic activity and energy metabolism influences over neuronal excitability.

Leite et al. (2002) identified a reduced amount of neuronal nitric oxide synthase (nNOS) in hippocampal sections (CA1–CA4) of samples from patients with MTLE and HS by immunohistochemistry (IHC) and western blot (WB) experiments. Nitric oxide impacts the neuronal membrane potential by changing the dynamics of neurotransmitter release and ion channels, phenomena that alter cell excitability (Leite et al., 2002; Fukata and Fukata, 2017). The tissues were obtained from patients with pathological signs of HS evaluated with high-resolution magnetic resonance imaging (MRI), and the controls were obtained from the autopsies of patients with no neurological conditions (Leite et al., 2002). This study was hypothesis-driven; its main purpose was to describe nNOS expression in the hippocampus of patients with MTLE and how it could be related to disease mechanisms.

The first evidence of changes in neuronal plasticity and the axonal organization at the protein level was published by Czech et al. (2004). A study of the middle segment of the hippocampal body from patients with drug-resistant MTLE (rMTLE) associated with typical imaging and pathological features of HS, compared with autopsy material, was conducted by 2DE (two-dimension electrophoresis), and spots were then analyzed by a MALDI-TOF PMF (Matrix-Assisted Laser Desorption Ionization Time of Flight Mass Spectrometry Peptide Mass Fingerprint) and MALDI LIFT-TOF/TOF MS/MS (matrix-assisted laser desorption/ionization time-of-flight/time-of-flight mass spectrometer). The researchers identified 13 different spots, including CRMP-2 at 65 and 55 kDa. The authors suggested that the expression of the two isoforms represents alternative splicing forms of post-translational modifications (PTMs; Czech et al., 2004). CRMP-2 is related to axonal outgrowth and neuronal polarity, which guides axonal pathfinding and is essential to brain plasticity (Babb et al., 1991; Isokawa et al., 1993; Blümcke et al., 1999; Furtinger et al., 2001; Gorter and Lopes da Silva, 2002). Therefore, reduced CRMP-2 could induce aberrant axonal reorganization and be related to HS. Recent studies have shown a relationship between elevated CRMP-2 and epilepsy, including the usage of lacosamide, a CRMP-2 antagonist, in the treatment of refractory partial epilepsy seizures (Wilson and Khanna, 2015; Keren-Aviram et al., 2018).

After the previous study (Czech et al., 2004), the same group analyzed hippocampal samples from patients with rMTLE and HS using 2DE followed by MS, comparing them with *postmortem* controls; they identified a reduction ACOT in patients (Yang et al., 2004). ACOT regulates intracellular levels of coenzyme A (CoASH), acyl-CoA, and free fatty acids (Yamada et al., 1999). These molecules are involved in fatty acid metabolism, influencing the electrochemical gradient by a cascade reaction (Yamada et al., 1999; Yang et al., 2004; Sousa et al., 2018). Another study from the same group revealed altered expression of ApoA-I in the hippocampus from patients with MTLE compared with autopsy controls. However, this change seems to be due to plasma extravasates and partly by gliosis, as shown by IHC analysis (Yang et al., 2005).

Subsequently, a pilot study using 2DE followed by MALDI-TOF/TOF MS compared the proteomic profile of a biopsy specimen of the human temporal lobe obtained from a patient with rMTLE to an autopsy specimen of the same brain region, from a patient with no prior history of neurodegenerative conditions (He et al., 2006). Enzymes and structural proteins represented the majority of the identified proteins, while channel and regulator proteins were rare. The neurotransmitter metabolic enzymes, such as DDAH1, are reduced in TLE patients; these changes could indirectly lead to alterations in the homeostasis and central functions of nitric oxide, like neuronal plasticity. Another enzyme that is decreased, glutamate-ammonia ligase (glutamine synthase), could be related to the extracellular accumulation of glutamate and excitotoxicity (Eid et al., 2004; He et al., 2006). Meanwhile, decreased aspartate aminotransferase and the GABA catabolic enzyme (4-aminobutyrate transaminase) would reduce the metabolism of glutamate and the catabolism of GABA, leading to the perturbation of a wide range of synaptic processes (He et al., 2006). Decreased UNG and SIR2 levels were also identified in the patient and could lead to neuronal death by impairing oxidative DNA damage repair (Rose et al., 2003; Kruman et al., 2004; He et al., 2006). Also, the cytoprotective protein CRYAB is associated with the tubulin cytoskeleton and could be related to some neurodegenerative conditions and TLE (He et al., 2006; Xi et al., 2006). Moreover, researchers have suggested that decreased levels of neurofilament three and septins are related to neuronal cytoskeleton damage: these changes impair cytoskeleton remodeling and tissue organization and function (Perez-Olle et al., 2004; Spiliotis, 2006; Xi et al., 2006). Besides, reduced DPYSL2 could also impair tissue organization due to abnormalities in axon guidance and neural growth cone collapse. A decrease in the level of enzymes related to glycolysis and tricarboxylic acids, such as ALDOA, PGK1, MDH1, IDH3A, LDH, and TPI1, could reduce the cellular energy supply (He et al., 2006). Despite the identified alterations, He et al. (2006) only analyzed one patient and one control, which makes it very difficult to relate these findings to the disease mechanism. The use of *postmortem* tissue as a control—with no description of the histological features—also makes it difficult to assess the validity of the results.

Another study has also described several proteins related to cytoskeleton function and tissue structure from patients with rMTLE compared with autopsy tissue from patients with no history of brain disease (Yang et al., 2006). In that work, 2DE and MALDI-TOF spectrometry identified reduced levels of α-1-tubulin and β-tubulin in two and three spots, respectively. Tubulin β-5 chain, ezrin, and the anchoring protein vinculin were increased in the patient samples. Profilin II and neuronal tropomodulin were decreased, while the presence of β-actin was not identified in the samples. The absence of tropomodulin can reduce neuronal death and affect the cytoskeleton integrity of those patients (Yang et al., 2006). The authors also described the alteration of oxidative stress proteins: they observed an increase in PRDX6 and a decrease in PRDX3, changes that were associated with reactive oxygen species (ROS) attacks and production, respectively, in MTLE patients. This protein responds to oxidative stress by modulating cytoskeletal dynamics (Wang et al., 2003; Yang et al., 2006). Reduced levels of chaperone 1 and T complex protein were observed in the same patient samples. T complex protein facilitates tubulin protein folding, while chaperone is proportionally involved in the cytoskeleton development level (Yang et al., 2006). Some reduced synaptic proteins were also associated with cytoskeletal mechanisms in MTLE patients. Low levels of MAPKK1 impairs the phosphorylation activity of cytoskeleton proteins, changing their functionality. SYTI and SCNA are substrates for the mitogen-activated protein kinase (MAPK) pathway. Their reduction in MTLE patients can regulate neurotransmitter release and transport and the formation of filamentous aggregates, causing synaptic deficits and cytoskeletal abnormalities (Yang et al., 2006). However, these alterations in cytoskeleton proteins could also be related to the gliosis and neuronal loss characteristic of HS; also, this study did not evaluate the histopathological features of the analyzed tissue.

The same MALDI-TOF technique was used to evaluate the untargeted proteomic profile of hippocampal samples from MTLE patients (Persike et al., 2012) compared with hippocampal tissue from autopsy controls. Overall, the authors identified nine altered proteins associated with metabolic disturbance and oxidative damage. There were increased levels of ALB, HSP70, DPYSL2, MBP, ATP6V1A, and the DLAT complex. There were decreased levels of SPTAN isoform 3, and two proteins were found only in samples from patients: GSTP1 and PARK7 (Persike et al., 2012).

In an elegant study performed in 2014, MALDI mass spectrometry imaging (MSI) was used to examine tissue protein from specific microdissected layers of the mesial temporal structures from patients with MTLE and autopsy controls (Mériaux et al., 2014). Histological observation of the hippocampus indicated HS with a localized loss of neurons in the CA1 and the granular layer of the DG. Due to the putative role of the DG in regulating the entrance of excitatory/inhibitory nerve signals in the hippocampus and its influences on the balance of the limbic system, the authors performed an MSI experiment using protein extracts from specific layers of the DG. They analyzed the data using hierarchical unsupervised clustering and reconstruction of the areas with different profiles (Hsu, 2007; Mériaux et al., 2014). These authors were the first to identify specific peptides in different layers of the DG. In the granular layer, there were galanin and neurokinin B; in the hilar layer, there was the C-terminally truncated fragment NPY(1–30); and in the molecular layer, they found somatostatin 1. Remarkably, these results are in accordance with previous findings, indicating that NYP and somatostatin are present in DG GABAergic interneurons, and the upregulation of NYP and its receptor in the DG is related to the seizure-induced neurogenesis (Furtinger et al., 2001; Kokaia, 2011; Gøtzsche et al., 2012; Mériaux et al., 2014). Somatostatin is a critical neuromodulator in the DG, and its levels are sensitive to excitotoxicity. Also, Mériaux et al. (2014) extracted proteins of 20 μm of microdissected hippocampal tissue sections and performed in-gel digestion and analysis by nanoLC-LTQ MS/MS (nano Liquid Chromatography Linear Trap Quadrupole in tandem Mass Spectrometry). Among the proteins with altered expression in the hippocampus of patients with MTLE, the authors found LIGI1, a protein involved with a hereditary form of NTLE, autosomal dominant partial epilepsy with auditory features (ADPEAF; Ottman et al., 2004).

Furthermore, researchers have found sex-specific proteins, such as tumor suppressor proteins, hormones (FSTL4 and BPIFB1), neurite outgrowth proteins (SRGP3, GPRIN3), and proteins implicated in Alzheimer’s disease (APP) in male patients with MTLE, whereas female patients had a specific signature for proteins involved in brain synaptic plasticity (OPTN, OPALIN, and LIN7C), odorant receptors, growth factors (SESN1, IGF1R, BMPR1A, IRF-2, and ABI1), and actin-associated cytoskeleton proteins (cortactin, APS2; Mériaux et al., 2014). Label-free quantification confirmed the presence of sex-specific neuropeptide precursors and their receptors. In male patients with MTLE, there were secretogranin-1, secretogranin-2, galanin, and appetite-regulated hormone isoform 2, and angiotensinogen, neuroendocrine convertase 2, NPY type 2 receptor, vasopressin, and the mu-type opioid variant receptors. In female patients, they identified pro-enkephalin B and growth hormone 2, and VIP and leptin receptors. Furthermore, there were sex-specific patterns for endocrine hormones and their receptors: steroid hormone and FSH receptors (Follicle-stimulating hormone) in male patients, and growth hormone 2 (GH2) and LHRH receptors (Luteinizing hormone-releasing hormone) in females with MTLE (Mériaux et al., 2014).

Researchers recently used neocortex samples obtained from patients who underwent cortical resections to treat refractory seizures. These neocortical tissues displayed high spike frequency during electroencephalography (EEG) monitoring, and their proteomic findings were compared with nearby tissue with no or low spike frequency. This interesting approach made it possible to compare protein expression in tissue from the same patient, thus decreasing the impact of individual variability (Keren-Aviram et al., 2018). The authors used a 2D-DIGE (two-dimensional difference gel electrophoresis) approach followed by LC/MS and an in-line microfluidic Chip LC. Eight proteins were upregulated in the high-spiking regions (SNCA, STMN1, UGP2, DSP, CA1, PRDX2, SYN2, and DPYSL2), and 10 were downregulated in the same regions (GFAP, HNRNPK, CPNE6, CRYAB, GNAO1, PHYHIP, HNRPDL, ALDH2, GAPDH, and LASP1; Keren-Aviram et al., 2018). By using specific histological staining, the authors concluded that most of the upregulated proteins were found predominantly in the high spiking neocortex’s blood vessels. Thus, they interpreted the finding of a highly expressed erythrocyte protein cluster as a part of the increased vascularity in high-spiking samples. The process of angiogenesis has been associated with brain injury and increased blood-brain barrier (BBB) permeability in TLE, suggesting that this process may be related to metabolic changes and increased cortical activity (Greenberg and Jin, 2005; Papageorgiou et al., 2011; Visanji et al., 2012; Keren-Aviram et al., 2018). However, it was not possible to indicate whether the vascular changes cause epileptic activity or result from increased activity. Also, researchers have identified changes in adherens junction proteins, which can be involved in blood vessels or the BBB.

Several other abnormally regulated pathways have also been found in epilepsy, such as “acute phase response signaling,” “Semaphorin signaling in neurons,” and “axonal guidance signaling,” indicating a high degree of changes in synaptic plasticity related to the epileptic neocortex. The identification of these pathways specifies the presence of CRMP isoforms, specially CRMP-2, which was upregulated in high-spike tissues. Moreover, GFAP, 50 kDa, total nuclear GFAP, and CRYAB were decreased in the neocortical tissues with high-spike frequencies. These results were unexpected because these proteins have been reported to be upregulated in neocortical epileptic tissue (Oberheim et al., 2008; Keren-Aviram et al., 2018). However, as previously discussed, He et al. (2006) also identified reduced CRYAB. CRYAB regulates GFAP assembly and it is strongly induced in reactive astrocytes. It plays a cytoprotective role with an anti-inflammatory and an anti-apoptotic function in those cells (Che et al., 2001; Ousman et al., 2007; Hagemann et al., 2009; Sarnat and Flores-Sarnat, 2009). Keren-Aviram et al. (2018) hypothesized that the co-reduction may be related to CRYAB–GFAP complex formation, reduced reactive astrocytes, or astrocyte apoptosis due to the lack of CRYAB. Furthermore, they showed that another candidate for the astrocyte marker, ALDH1L1, was also decreased. For all these results, they suggested that GFAP-positive astrocytes may be reduced and related to epilepsy, but the nature of the changes in these cells is unclear. Besides those patterns, they also identified significant individual proteins. In high-spiking neurons, they found downregulation of GNAO1, LIM, and LASP1, which may also be related to the astrocyte origin and plenty of other functions, and upregulation of SNCA, SYN2, and STMN1 which may be linked to neurite sprouting and synapse remodeling, because they interact with cytoskeletal proteins in the nerve growth cone and microtubule regulation (Keren-Aviram et al., 2018).

A recent study with histopathology and IHC showed reduced levels of mitochondrial ATP synthase (ATP5B) in all hippocampal regions of patients with MTLE and HS compared with *postmortem* control hippocampal tissue. The decreased level of ATP5B might be associated with neuronal cell maintenance (Mota et al., 2019). ATPases maintain an adequate neuronal transmembrane electrical potential, and, consequently, dissipate ionic transients and protect brain functions (Yang et al., 2004).

Furthermore, PTMs can lead to protein dysfunction and affect the phenotype. A recent study evaluated the cycling and disruptions of two PTM enzymes and their effect on hippocampal tissues from an animal model and patients with rMTLE compared with age-matched *postmortem* hippocampus as controls (Sánchez et al., 2019). Using HPLC and MS/MS analysis, electrophysiology, WB, pharmacological tests, immunofluorescence, and animal MRI, the authors found that the absence of OGA increases OGlcNacetylation modification of the tissue, reducing the duration of seizures and epileptic spike events. They concluded that OGA can be a potential therapeutic target for seizure control (Sánchez et al., 2019).

Also, the phosphorylation of amino acid residues in proteins with the potential to lead to changes in the functioning of neurons has been described in epilepsy. A work evaluating differences between patients with rMTLE associated with HS (MTLE-HS), who may or may not present a clinical sign called ictal fear (IF), showed a difference in the phosphorylation of a residue belonging to a subunit of the AMPA receptor, GluA1-Ser845. The authors used hippocampal and amygdalar homogenates from surgeries and determined the phosphorylation levels and the total amount of target proteins using WB, and, for the detection of proteins, selective antibodies. Thus, the authors demonstrated that patients with MTLE and IF had a significant decrease in p-GluA1-Ser845 in the anterior portion of the hippocampus and decreased GluA1 subunit levels in the amygdala ipsilateral to the HS compared with patients without IF (Leal et al., 2020). Of note, the authors did not use controls: they analyzed only the epileptic tissue.

### Malformations of Cortical Development

Another important cause of refractory epilepsy is abnormal cortical development. These structural abnormalities are caused by disturbances during the development of the cortical layers, networks, and columns (Palmini et al., 1992). Many studies addressing the anatomical, molecular and electrophysiological characteristics of these malformations have been performed over the past two decades; however, it is still unclear how the size and location of the abnormalities are correlated with the severity of the seizure (Kilb et al., 2011; Schwartzkroin and Wenzel, 2012; Luhmann et al., 2014). Although abnormal cortical development is an important cause of focal epilepsy, proteomic studies exploring their mechanisms and biological responses are still scarce. Among the most common types of cortical malformations are focal cortical dysplasia (FCD), which is commonly found in children and is one of the main causes of medically refractory seizures in this group of patients with focal seizures (Blümcke et al., 2011).

A recent study evaluated brain tissue from patients with childhood cortical dysplasia (CCD) compared with surgical control tissue from patients with traumatic intracranial hypertension (TIH), by using iTRAQ (Isobaric tag for relative and absolute quantitation) labeling and MS/MS to perform a screening of the differentially expressed (DE) proteins (Qin et al., 2017). The authors identified 153 DE proteins in the CCD tissues and further validated their findings with WB and IHC. Through gene ontology, these were classified mainly into groups facilitating catalytic activity, binding, transporter activity, enzyme regulation, and molecule-structuring activity. Among the upregulated proteins, they identified FSCN1, CRMP1, NDRG1, DPYSL5, MAP4, and FABP3, while PRDX6 and PSAP were downregulated. This was the first report of these proteins being associated with CCD. The increased levels of the proteins FSCN1, CRMP1, and MAP4 may be involved with abnormal neuron migration, neurite outgrowth, abnormal dendrite orientation, and the later involved with microtubules movement (Bast et al., 2006; Yamashita et al., 2006; Tokuraku et al., 2007; Higurashi et al., 2012; Wu et al., 2014; Qin et al., 2017). NDRG1 was also upregulated in CCD tissues and seemed to be part of a complex mechanism involved with reactive oligodendroglial hyperplasia (Shepherd et al., 2013; Qin et al., 2017). Meanwhile, decreased PRDX6 levels are associated with oxidative stress, a mechanism highly associated with brain damage and epilepsy, as discussed previously (Singh and Trevick, 2016; Qin et al., 2017). PSAP has been associated with animal models with neuroprotection, suggesting that its decrease could have an important role in neurotoxicity (Nabeka et al., 2014; Qin et al., 2017).

Tuberous sclerosis (TSC) is a multisystem autosomal dominant condition with a prevalence of 1 in 6,000 live births. TSC shares many histopathologic features with FCD, and most patients with the disease present mutations in *TSC1* or *TSC2*. The classical TSC lesions are hamartomas, but neurologic issues represent some of the most relevant clinical complications (Saxena and Sampson, 2015; Canevini et al., 2018). Genetic mutations in both genes result in the abnormal activation of the mammalian target of rapamycin (mTOR) pathway. This mTOR overactivity may lead to aberrant migration and orientation of neuronal cells that, consequently, drive atypical cortical lamination and dendritic arborization (Canevini et al., 2018). Epilepsy is the most prevalent clinical manifestation of TSC, affecting 85% of patients; infantile spasms are frequently accompanied by other seizure types, resulting in 75% of cases with pharmacoresistant epilepsy (Saxena and Sampson, 2015; Curatolo et al., 2018).

A study characterized overexpression of four microRNAs from cortical tubers resected from patients with a mutation in *TSC1* and *TSC2*. The authors compared epileptogenic tuberous tissue with adjacent non-tuberous tissue using quantitative LC-MS/MS proteomics to assess alterations in the abundance of the microRNA-targeted proteins; they identified significant repression in the expression of these proteins. The set of transcripts that were repressed has been associated with synaptic signaling and inflammation. Among these repressed proteins, they identified hamartin, which is a product of the *TSC1* gene. Based on the study results and data from the literature, the authors proposed a possible role for these aberrantly expressed microRNAs, especially miRs-23a and miRs-34a, in the tuber pathology. Furthermore, aberrantly expressed microRNAs are likely involved with alterations in synaptic density and loss of neuronal elements of TSC patients (Dombkowski et al., 2016).

A very recent study (Liu et al., 2020) reported mutation analyses, clinical features, and tissue proteomic profiles of three patients with epilepsy caused by truncating *TSC1* mutations. The authors used a DIA (Data-independent acquisition) workflow to explore the proteomic profile of these patients compared with control tissue from craniocerebral trauma surgery and found downregulation of ARFGEF2, which has been associated with intellectual disability and abnormal neuronal migration (Hong et al., 2018; Liu et al., 2020). DCX upregulation may also be involved with intellectual disability, abnormal neuronal localization, epilepsy (des Portes et al., 1998; Lee et al., 2003; Liu et al., 2020), and decreased levels of ATG12, a protein related to autophagy (Wang et al., 2019). Moreover, Liu et al. (2020) identified decreased levels of K^+^ channel proteins (KCNA2 and KCNAB2) in the *TSC1* mutation group compared with the controls; these changes have been associated with epilepsy due to the proteins’ role in membrane excitability (Jan and Jan, 1997; Liu et al., 2020). In summary, through the enrichment gene ontology analysis, the researchers found that DE proteins were mainly the components of synaptic membranes; the biological processes that were highly enriched were amino acid metabolism and the molecular functions related to antioxidant activity, ligase activity, and tetrapyrrole binding, which had an especially high number of DE proteins. Taken together, these data suggest that TSC mutations or alterations in patients’ synaptic proteins may affect the information transmission in the cells, and changes in patients’ amino acid metabolism can be a new mechanism to be explored in the TSC complex context (Liu et al., 2020).

Notably, despite the many efforts to explore and describe epilepsy-related changes at the protein level, most published neuroproteomics studies are limited by technical constraints and have mainly used low in-depth coverage techniques. The first published neuroproteomics study, from the early 2000s, used predominantly two-dimensional polyacrylamide gel electrophoresis (2D-PAGE). Unfortunately, besides being time-consuming, 2D-PAGE requires large amounts of input material, it is labor-intensive, and it is limited to detecting low-abundance and hydrophobic proteins (Zhang et al., 2014; Yokota, 2019). It was only around 2014 that studies using LC-MS started to appear in the epilepsy field; this technique has allowed for more extended coverage of the proteome and enhanced dynamic range and sensitivity. Another challenge, specific to studies with human specimens, is the limitations imposed by the use of *postmortem* tissue as controls, and the lack of age-matched subjects. As mentioned previously, this approach may introduce confounding factors in data interpretation due to the delay in tissue collection and processing, which directly affects the rate of protein degradation (ElHajj et al., 2016; Ferreira et al., 2018). Hence, studies in animal models may provide relevant information that can complement the results obtained with human samples by overcoming the lack of proper controls for human tissue (Becker, 2018).

## Proteomic Studies in Tissue From Animal Models of Epilepsy

Animal models, mainly rodents, which present physiological characteristics that mimic human epilepsy, are often used to study the mechanisms and possible therapeutic strategies for epilepsy. They can be divided into two main categories: acquired models, usually induced through a stimulus like an injury trauma, the use of chemo convulsant, and electrical stimulation of brain regions; or genetic models caused by genetic modifications that lead to spontaneous recurrent seizures (Löscher, 2017). Here, we will review the main findings of proteomic studies using tissue from animal models of epilepsy.

### Acquired Models

#### Pilocarpine

The pilocarpine models of MTLE are based on the induction of a *status epilepticus* (SE, a seizure that lasts at least 5 min uninterrupted), and its physiological features reflect pathologic patterns of human MTLE, with the occurrence of spontaneously recurrent seizures and interictal activity patterns (Cavalheiro et al., 1991; Sharma et al., 2007; Curia et al., 2008). Pilocarpine is a drug that acts as a muscarinic acetylcholine receptor partial agonist, specifically the M1 receptor. It induces long-term epileptic activity and neuronal damage with structural alterations and neuronal death mainly in the limbic regions (Cavalheiro et al., 1991; Curia et al., 2008). Using 2DE-based proteomics to evaluate changes in the hippocampal tissue from pilocarpine-treated animals 2 days after SE (Greene et al., 2007), the most significant difference was the upregulation of HSP27, which is linked with responses to cellular stress and could also be performing a neuroprotective role in these cells (Kalwy et al., 2003; Arimura et al., 2004; Czech et al., 2004). DRP-2 was also changed: it was downregulated in the hippocampus of epileptic animals. This protein is related to the development of newborn neurons and axon growth, which are directly involved with neuronal development. Furthermore, there was elevated expression of α-tubulin, which is also related to axon outgrowth and microtubule functions, indicating that structural abnormalities are a significant contributing factor to epileptogenesis in this model. Moreover, an observed increase in DHPR in the hippocampus from pilocarpine-treated rats could result in elevated BH_4_ levels (Cho et al., 1999), contributing to neuronal damage following SE. The authors suggested it as a possible therapeutic target (Greene et al., 2007).

Another study using hippocampal tissue from a pilocarpine-induced model evaluated two-time points, 12 h (acute) and 72 h (latent) after SE compared with controls; the authors identified 57 DE proteins using 2DE coupled with MALDI-MS and MS/MS (Liu et al., 2008). This study demonstrated that abnormal expression of structural (actins and tubulins) and mitochondrial (PKM2, ATP5A1, ATP5B, VDAC2, HSP60, and others) proteins and impairment in synaptic transmission and plasticity (HOMER2, SYN2, and SNAP25) could be involved with the pathological mechanisms underlying epilepsy. Among these mechanisms, the authors suggested that these alterations could lead to modifications in information storage, long-term plasticity, structural alterations of neural circuits, synaptic impairment, inadequate energy supply, changes in ion homeostasis, apoptosis, oxidative stress, and protein misfolding, which could be related to seizure-induced neuronal damage and neuroprotection (Liu et al., 2008).

Li et al. (2010) analyzed the DG from a pilocarpine model of epilepsy using 2DE combined with LC-MS/MS to identify the protein abundance and phosphorylation state of these neurons. The authors reported eight functional protein categories: metabolism, synaptic, energy, structural, stress response, apoptosis, transcription, and growth. Among the structural proteins, β-actin was downregulated and profilin-1 and vimentin were upregulated. From synaptic proteins, α-synuclein and UCH-L1 were upregulated. The researchers also identified several proteins involved with metabolism, such as carbonic anhydrase II, which was enhanced phosphorylated in the epileptic hippocampus. Another protein that was enhanced and phosphorylated by SE was NSE2, a marker of neuronal damage. There was also a 141% increase in the expression of the cathepsin D. The cell stress response was exacerbated through increased expression of PRDX6, a potent antioxidant, and increased expression of the HSP27 and CRYAB (Li et al., 2010).

A more recent study used the pilocarpine model to analyze the right and left hippocampus using 2DE and MALDI-TOF-MS (Sadeghi et al., 2017). Lateralization has been reported to be very relevant, especially with the predominance of the left hemisphere in human epilepsy (Gatzonis et al., 2002). When analyzing only the control hippocampus (healthy) to evaluate changes in both hemispheres, the researchers found a significant increase in the expression of the proteins DJ-1, SPR, BASP1, α-Inx, AADC, CLCA, and CLCB in the right hippocampus. These proteins are related to dopamine synthesis. Overall, the study revealed a decrease in the proteins involved in dopamine pathways (DJ-1, SPR, AADC, β-synuclein, BASP1, CLCA, CLCB, SNAP25, α-Inx, and STMN1) in the pilocarpine model. Also, there was an increase in polyamine regulatory enzymes (mitochondrial ornithine transporter and ODC), which were higher in the left epileptic hippocampal tissue. This lateralization could lead to a lower seizure threshold in the left hemisphere in the epilepsy context (Sadeghi et al., 2017).

In a very recent study performed by our group in 2020, we employed the pilocarpine model to broadly assess changes in the proteome and transcriptome of Wistar rats (Canto et al., 2020). We isolated different regions of the hippocampal formation, DG and CA3, as well as different areas (dorsal and ventral), using laser microdissection and determined their protein and transcriptional profiles—the transcriptomic study used next-generation sequencing, while the proteomic study used a label-free mass spectrometry approach. After integrating the data between the two molecular techniques, we found several pathways suggestive of enhanced epileptogenesis in the CA3 compared with the DG. Also, we described a complex network of cellular pathways that contribute to the generation of epileptogenesis in different regions and areas of the hippocampal formation, such as: (i) upregulation in pathways related to inflammation and immune response; (ii) downregulation in calcium/calmodulin-dependent protein kinases (CAMKs); and (iii) abnormal regulation of ion channels, such as some voltage-gated K^+^ and voltage-gated Na^+^ channels. We also found upregulation of genes from pathways associated with neurogenesis in the DG (*Notch1-201* and *Ephb6-201*) and the LRRK2 (Leucine-rich repeat kinase 2) pathway in the dorsal region of the DG. By contrast, there was a downregulation in the WNT signaling pathway in the CA3. These findings also highlight the heterogeneity in the transcriptome and proteome of different hippocampal regions and areas (Canto et al., 2020).

#### Kainic Acid

The intra-hippocampal administration of kainic acid (KA) is another animal model for MTLE; it is widely used in the study of the histopathological changes occurring in MTS because many of the characteristic changes found in human MTS are seen in this model (Bouilleret et al., 1999; Heinrich et al., 2011). Thus, it is believed that characterizing the proteomes of these animals can provide information about the cellular mechanisms involved in the establishment of epileptogenesis and cellular events triggered by the establishment and progression of the disease (Kim et al., 2004). Many studies have used this approach to identify determining factors for the recurrent onset of seizures and the mesial lesions commonly found in patients with MTLE. With this focus, a study evaluated the hippocampal proteome of KA-induced mice using high-resolution Orbitrap LC-MS/MS combined with label-free quantification at three different times after the injection of the chemical (1, 3, and 30 days), with NaCl-injected mice as controls, to evaluate molecular patterns that may provide information about the evolution of the disease (Bitsika et al., 2016). The authors reported decreased expression of proteins related to axonal regeneration and neuroplasticity, mainly in the chronic phase of the disease (30 days after KA injection). Downregulated proteins such as neurochondrin, Homer1, Ataxin-10, and Neurabin-2 suggest dysfunctions in neuroplasticity. By contrast, changes in MAP2, tau (Mapt), and BRSK1 may characterize the neurodegeneration profile observed in this model. Therefore, these changes could trigger characteristic histopathological findings in MTLE, such as neuronal loss observed in hippocampal regions including the DG (Bitsika et al., 2016).

Bitsika et al. (2016) also reported increased expression of other proteins related to inflammatory processes, such as a link with the activation of microglia/astrocyte, which extends from the stage of development of the disease (3 days after KA injection) to the chronic phase. This process was characterized by elevated expression of proteins linked to neuroprotection resulting from inflammatory processes, such as clusterin and gelsolin, as well as pro-inflammatory cytokines such as C4B, α-2-macroglobulin, CD44, and some integrin receptors in T cells (Bitsika et al., 2016).

In addition to the quantitative analysis of protein expression, it is also possible to analyze PTMs, which may provide information regarding protein activity (Kumar and Prabhakar, 2008). In this context, a study published in 2014 showed downregulation in 12 phosphorylation sites of Nav1.2 channels in rats with acute seizures induced by systemic KA injection, using the immunopurification of Nav1.2 and subsequent gel-based proteomics by nano-LC tandem LTQ-FT MS (Baek et al., 2014). One of the target regions for the insertion of a phosphate group identified by this approach, the ID I-II linker, when downregulated, leads to an increase in ionic currents, a phenomenon possibly related to the onset of seizures and the subsequent epileptogenesis. Besides, increased methylation in some arginine residues (R472, R563, and R570) from these channels, which can contribute to the development of seizures, has also been described (Baek et al., 2014).

Mitochondrial protein PTMs also play an important role in overall cellular functioning: These modifications participate in the regulation of central metabolism pathways, such as those related to bioenergetics. It is well known that epileptogenesis and seizure development is closely associated with mitochondrial dysfunctions because this organelle plays a determining role in the production of ATP and the control of oxidative damage (Kovac et al., 2013; Rowley and Patel, 2013). Therefore, a study sought to demonstrate, through WB analysis of the mitochondrial isolate from hippocampal homogenates, which PTMs have great relevance to cellular processes that culminate in the establishment of recurrent epileptic seizures (Gano et al., 2018). The authors reported a decrease in SIRT3, a protein responsible for NAD^+^-dependent deacetylation of mitochondrial proteins, bioenergetics, and antioxidant mechanisms, especially in the chronic phase of the model. This reduction was correlated with some of the main characteristic cellular changes of the disease, including alterations in bioenergetics, antioxidant pathways, and epileptogenesis. Thus, as a result of this finding, the acetylation levels of mitochondrial proteins would appear to be increased. Also, there were other alterations, such as a decrease in NAMPT, with an effect on NAD^+^ levels. Also, increased acetylation of MnSOD and IDH2 may lead to impairment in the oxidative equilibrium of hippocampal cells with the potential to contribute to the commonly observed neurodegeneration pattern in MTLE (Gano et al., 2018).

#### Pentylenetetrazol

Another biologically active molecule that can generate acute or chronic seizures is pentylenetetrazol (PTZ; Takechi et al., 2012). The pro-convulsive action of PTZ is due to its antagonistic activity on GABA_A_ receptors, thus generating a decrease in the inhibitory effect produced by GABA when it binds to its receptors (Ramanjaneyulu and Ticku, 1984). Although this model has been studied since the mid-1960s, the process of elucidating the proteins involved in the mechanism of PTZ-induced seizures is not yet complete (Löscher, 2011). However, it has already been shown that increased susceptibility to seizures through the administration of this molecule is linked to increased expression of tissue plasminogen activator (tPA) and some structural proteins, such as MAP1B and GAP43, in hippocampal tissue (Junker et al., 2005). Also, the authors demonstrated, through a gel-based proteomic analysis followed by a MALDI-TOF approach using hippocampal tissues from rats induced with PTZ that molecular modifications—possibly linked to PTM—of the Rieske iron-sulfur protein may be related to the seizure development process in this model. Given that the Rieske iron-sulfur protein participates in the protein structure of the mitochondrial cytochrome *bc*_1_ complex, it was proposed that changes in its activity are characterized by the decrease in the energetic state of the cells—a development with the potential to facilitate the occurrence of seizures and leading to some histopathological findings, such as the damage in the CA1 subfield of the hippocampal formation (Junker et al., 2005).

#### Electrical Stimulation

The electrical stimulation approach of specific neuronal pathways also has great importance in the induction of epilepsy in animal models, mainly rodents. This method retains considerable relevance in the scope of animal models due to the generation of cell lesions similar to those found in human MTLE, with less extra-hippocampal injury (Norwood et al., 2010). Despite being a well-characterized model, the molecular mechanisms that lead animals to recurrent seizures are still poorly understood (Norwood et al., 2010; Will et al., 2013). In this sense, to offer molecular bases for such pathophysiological changes, a study published in 2017 showed that the hippocampal tissue and the parahippocampal cortex of Sprague Dawley rats were induced by stimulation of the right anterior basolateral region of the amygdala for proteomic analysis by LC-MS/MS at different stages of the disease (Keck et al., 2018). In the hippocampus, the authors identified 121 DE proteins 2 days after SE, 276 DE proteins after 10 days, and 14 DE proteins after 8 weeks. In the parahippocampal cortex, there were 218, 419, and 223 DE proteins 2 days, 10 days, and 8 weeks, respectively, after SE. Therefore, it was possible to describe temporal changes in molecular profiles that were possibly linked to the histopathophysiological changes observed in the animals. These profiles displayed, in the hippocampus, enriched pathways commonly linked to the regulation of cell death (14-3-3 and HIPPO signaling), cellular plasticity (cytoskeletal dynamics and axonal guidance), and carbohydrate and amino acid metabolism (Keck et al., 2018). More recently, our group analyzed the proteome of the DG from perforant pathway–stimulated rats by LC-MS/MS; we identified different proteomic profiles for the DG layers (granular and molecular) and regions (ventral and dorsal; do Canto et al., 2020). In all layers and regions, there were two enriched pathways that are important for determining the molecular bases for epileptogenesis and the cellular changes observed: inflammation and energy metabolism. The enriched inflammation pathways (immune response-CRTH2 signaling in Th2 cells, IL-16 signaling pathway, and others) contained abnormally regulated proteins such as PKC and 14-3-3 proteins. Those related to bioenergetics (citric acid cycle, gluconeogenesis, and amino acid metabolism) exhibited several downregulated proteins, pointing to a dysfunction in the energy generation process of the cells. In addition to these pathways and proteins, others were linked to specific DG layers and regions. Proteins such as PARK7 and connexin 31/gap junction were differentially regulation in the ventral portion of the DG, and RACK1 expressed opposite differential regulation in the dorsal and ventral part of the DG (do Canto et al., 2020).

Due to the relevance of the inflammatory processes concerning human MTLE and animal models, some researchers have examined proteins closely linked to inflammatory pathways to determine their role in the main histopathological findings observed in the hippocampal tissue. In a study aiming to establish such a role, Walker et al. (2016) explored the proteome of the hippocampus and the parahippocampal cortex of rats induced with electrical stimulation of the right anterior basolateral nucleus of the amygdala was obtained by LC-MS/MS. The authors also evaluated the proteome in three stages of the disease—the early post-insult (2 days after SE), the latency phase (10 days after SE), and the chronic phase (8 weeks after SE)—to assess changes in the inflammatory profile as a function of disease progression. They showed an early induction for inflammatory pathways through the enrichment analyses of these two regions, with a sustained activity during the latency phase that preceded recurrent seizures. Moreover, in the phase where the disease is established (chronic phase), inflammatory pathways are predominant in the parahippocampal cortex compared with the hippocampus. Part of the inflammatory outcomes was related to the modulation of TLR (Toll-like receptors) signaling molecules, such as an increased expression of RPS27A, ITGB2, and ITGAM, mainly during the first two phases of the disease.

In addition to TLR signaling, some molecules related to purinergic receptors were found, such as P2RX7 upregulated in the hippocampus during epileptogenesis. These findings suggest that these receptors contribute to the maintenance of neuroinflammatory processes (Walker et al., 2016).

### Genetic Models

Among the animal models of epilepsy, genetically predisposed species display a similar pattern of spontaneous seizures and clinical phenotypes that is seen in some human epilepsy. In these models, the seizures occur spontaneously or in response to a stimulus (Löscher, 1984). The genetic models of absence epilepsy have been frequently studied. The Genetic Absence Epilepsy from Strasbourg (GAERS) and Wistar Albino Glaxo/Rijswijk (WAG/Rij) rats are widely used as rat models; they present behavioral and EEG characteristics that mimic the generalized human absence epilepsy (Depaulis and van Luijtelaar, 2006). Meanwhile, the BS/Orl and BR/Orl are genetic “in-mirror” mouse models (Chapouthier et al., 1998).

#### Absence Seizures

Absence seizures (AS) are a generalized type of epileptic seizures that frequently occur in children. AS are characterized by an abrupt loss of consciousness, motionless stare, and suspension of ongoing actions. The common hallmark of AS is bilaterally generalized synchronous “spike and wave” discharges (SWDs). It is broadly accepted that the alternation from healthy states to SWDs occurs after changes in corticothalamic connectivity, such as increasing excitatory connections between the cortex and the thalamus. The thalamic contribution to SWD triggering has been observed in *in vivo* studies (Maksimenko et al., 2017; Deeba et al., 2018). Searching for altered intracellular proteins in AS, a study using 2DE coupled with Nano-LC-ESI-MS/MS analyzed three brain structures from the GAERS rat model compared with non-epilepsy animals: the parietal cortex, thalamus, and hippocampus (Danış et al., 2011). The delta subunit of ATP synthase and the 14-3-3 zeta isoform were upregulated in the parietal cortex. Myelin basic proteins and macrophage migration inhibitory factor was upregulated in the thalamus and, in the hippocampus, the migration inhibitory factor was upregulated. At the same time, 0-β-2-globulin was downregulated. These findings indicate that, while intracellular proteins have not been seen as primarily responsible for neuronal excitability in seizures, they are relevant for maintaining neuronal functions and are linked to several mechanisms. Here are some examples: (i) the generation of energy through changes in ATP synthase; (ii) inflammatory responses represented by the macrophage inhibitory factor; (iii) ion channel and signal transduction with the alteration of 14-3-3 zeta and membrane potassium conductance; and (iv) abnormal expression of myelin basic protein and 0-β-2-globulin (Danış et al., 2011).

While Lagarrigue et al. (2012) aimed to develop a new proteomics workflow using MSI to detect possible disease markers, they used the BR/Orl and BS/Orl mouse models, compared them with the non-epileptic controls, and identified some interesting proteins. Among the findings, the SYN1 fragment was one of the most statistically significant fragments identified in the epileptic tissue. It was a potential marker of absence epilepsy because it is thought to control excitability (Garcia et al., 2004; Lagarrigue et al., 2012). The findings also identified DE of myelin basic protein, neurogranin, PCP4, ubiquitin, and thymosin β-4. These proteins have essential roles in the central nervous system and can contribute to the pathogenesis of the disease (Lagarrigue et al., 2012). Another study, using 2DE-MALDI-TOF MS, found two DE membrane proteins in the epileptic tissue compared with controls, namely 14-3-3 (upregulated) and the membrane-anchored protein GNBPs1 (downregulated). These data suggest that both of them can be important targets for the neuropharmacology studies of this disease (Yuce-Dursun et al., 2014).

#### Tuberous Sclerosis

There is interesting research that focuses on the role of abnormal proteins in animal models of TSC. A study published in 2016 aimed to explore the role of mTORC1 in regulating regional protein expression during disease and normal states used MS/MS combined with protein-protein interaction (PPI) analysis. The authors identified PARK7 protein as increased in dendrites and colocalized with post-synaptic density protein-95 (PSD95). To confirm these predicted results in the context of the disease state, they tested a *Tsc1* conditional knockout (cKO) mouse model and performed IHC. The *Tsc1*-cKO mouse model presents elevated mTORC1 activity due to TSC1 protein reduction, and it exhibits behavioral features of both epilepsy and autism. The mouse model results indicated that the expression of PARK7 increases in regions where the activity of mTORC1 is higher (Niere et al., 2016).

## Future Directions

In recent years, proteomic studies have become more advanced, mostly due to the incorporation of new technology, such as MS. These advances have led to increased accuracy and sensitivity, fast acquisition rates, high resolution, and the use of small input samples (Hosp and Mann, 2017). The variations detected in the proteome may result from different physiological states, differential gene expression, and regulation, or translational modifications (Ramadan et al., 2017; Li et al., 2019). Although neuroproteomics can help disentangle some of the brain’s biological complexity in normal and disease states, the results are limited by the disease complexity, variability in etiology, and brain areas analyzed.

These studies must account for the remarkable individual variation among human subjects and the intrinsic limitations in the characterization of physiological and, most importantly, disease-related phenotypes. Indeed, one of the most critical drawbacks identified in the neuroproteomics literature is the limited clinical characterization of the studied patients (Li et al., 2019). However, even with all the aforementioned constraints, systems biology combined with neuroproteomics analyses can lead to a new level of visualization and interpretation of the molecular information (Jaber et al., 2016a,b; Ramadan et al., 2017). Thus, generating new biological hypotheses about the molecular mechanisms of disease that can be further explored in depth in follow-up studies, especially in those with a multi-omics approach. In epilepsy, the most critical findings in proteomic studies have been the changes detected in structural proteins, especially those that comprise the cytoskeleton, oxidative stress, and energy metabolism—with an emphasis on the lipid metabolism identified especially in human studies; alterations of ion channels, inflammation, and synaptic proteins have been prevalent in most of the described studies (Figure 2). These changes could directly or indirectly affect the neurotransmission processes, which are mainly impaired in epilepsy. However, which mechanisms are more related to the cause or consequence of the disease remains unknown. Gliosis and extensive neuronal damage, as much as inflammatory responses, are frequently found in epilepsy patients and animal models. One of the greatest challenges when investigating the molecular mechanisms of epilepsy is to identify which abnormal biological pathways are the result of the histopathological changes that occur in the epileptic tissue and which ones are related to the mechanism leading to the lesions in the first place. Furthermore, it remains to be determined whether abnormally expressed proteins and enriched molecular pathways are indeed leading to seizures or maybe protecting against it.

The majority of the proteomic analyses of epilepsy are not hypothesis-driven (Ramadan et al., 2017); therefore, they have identified a broad set of biological mechanisms that can now guide future studies with defined, critical, and correct questions to be explored, such as a specific protein or PTM changes, or a dysregulated network involved with one particular pathological state. More important, future applications of more advanced technology in proteomic studies are essential to improve data quality and coverage and the acquisition of relevant biological information.

Furthermore, these protein identifications and quantifications can help researchers understand the disease progression and prognosis because some of the studies have analyzed the disease at various time points to better evaluate its biological effects. These findings can also, soon, contribute to the clinical diagnosis by being applied to the biomarker studies, helping predict and understand the drug-resistance mechanisms.

## Conclusion

Epilepsies represent a large group of heterogeneous conditions with complex molecular mechanisms. Neuroproteomic studies exploring different epilepsy types have made significant contributions, pointing to an array of biological processes that are involved in these conditions, especially energy metabolism, oxidative stress, inflammation, and excitatory imbalance. Proteomic analyses in epilepsy are useful because the analyzed tissue sample closely represents the pathogenic process involved in human epilepsies. However, the lack of an ideal experimental model that can faithfully reproduce the condition seen in patients has created difficulties in interpreting proteomic findings in animal models. At the same time, the use of human tissue samples can also be problematic because they are usually not available at a critical point in time when epileptogenesis actually occurs.

Furthermore, one cannot rely on human tissue to understand how the disease progresses over time. Another limitation of using tissue samples from patients is the remarkable individual variability among patients, a factor that may create confounding factors when interpreting the proteomic findings. It is clear that there are no simple solutions for the aforementioned limitations; thus, the field will significantly benefit from additional studies performed with large samples of well-characterized tissue so that the full array of proteomic changes occurring in epileptogenic abnormalities can be well characterized. Also, studies comparing the findings using similar proteomic techniques in different models and human tissue will be of great value in future studies.

## Author Contributions

All the authors contributed equally to the development, elaboration, and writing of this work. All authors contributed to the article and approved the submitted version.

## Conflict of Interest

The authors declare that the research was conducted in the absence of any commercial or financial relationships that could be construed as a potential conflict of interest.

## References

[B1] Al Diffalha S. Sexton K. C. Watson P. H. Grizzle W. E. (2019). The importance of human tissue bioresources in advancing biomedical research. Biopreserv. Biobank. 17, 209–212. 10.1089/bio.2019.0039 31188626 PMC7061295

[B2] Allone C. Lo Buono V. Corallo F. Pisani L. R. Pollicino P. Bramanti P.. (2017). Neuroimaging and cognitive functions in temporal lobe epilepsy: a review of the literature. J. Neurol. Sci. 381, 7–15. 10.1016/j.jns.2017.08.007 28991719

[B3] Arimura N. Menager C. Fukata Y. Kaibuchi K. (2004). Role of CRMP-2 in neuronal polarity. J. Neurobiol. 58, 34–47. 10.1002/neu.10269 14598368

[B4] Babb T. L. Kupfer W. R. Pretorius J. K. Crandall P. H. Levesque M. F. (1991). Synaptic reorganization by mossy fibers in human epileptic fascia dentata. Neuroscience 42, 351–363. 10.1016/0306-4522(91)90380-7 1716744

[B5] Baek J.-H. Rubinstein M. Scheuer T. Trimmer J. S. (2014). Reciprocal changes in phosphorylation and methylation of mammalian brain sodium channels in response to seizures. J. Biol. Chem. 289, 15363–15373. 10.1074/jbc.M114.562785 24737319 PMC4140893

[B6] Bast T. Ramantani G. Seitz A. Rating D. (2006). Focal cortical dysplasia: prevalence, clinical presentation and epilepsy in children and adults. Acta Neurol. Scand. 113, 72–81. 10.1111/j.1600-0404.2005.00555.x 16411966

[B7] Becker A. J. (2018). Review: animal models of acquired epilepsy: insights into mechanisms of human epileptogenesis. Neuropathol. Appl. Neurobiol. 44, 112–129. 10.1111/nan.12451 29130506

[B8] Bitsika V. Duveau V. Simon-Areces J. Mullen W. Roucard C. Makridakis M.. (2016). High-throughput LC-MS/MS proteomic analysis of a mouse model of mesiotemporal lobe epilepsy predicts microglial activation underlying disease development. J. Proteome Res. 15, 1546–1562. 10.1021/acs.jproteome.6b00003 27057777

[B9] Blair J. A. Wang C. Hernandez D. Siedlak S. L. Rodgers M. S. Achar R. K.. (2016). Individual case analysis of postmortem interval time on brain tissue preservation. PLoS One 11:e0151615. 10.1371/journal.pone.0151615 26982086 PMC4794172

[B10] Blümcke I. Beck H. Lie A. A. Wiestler O. D. (1999). Molecular neuropathology of human mesial temporal lobe epilepsy. Epilepsy Res. 36, 205–223. 10.1016/s0920-1211(99)00052-2 10515166

[B11] Blümcke I. Thom M. Aronica E. Armstrong D. D. Bartolomei F. Bernasconi A.. (2013). International consensus classification of hippocampal sclerosis in temporal lobe epilepsy: a task force report from the ILAE commission on diagnostic methods. Epilepsia 54, 1315–1329. 10.1111/epi.12220 23692496

[B12] Blümcke I. Thom M. Aronica E. Armstrong D. D. Vinters H. V. Palmini A.. (2011). The clinicopathologic spectrum of focal cortical dysplasias: a consensus classification proposed by an *ad hoc* Task Force of the ILAE Diagnostic Methods Commission1: the ILAE classification system of FCD. Epilepsia 52, 158–174. 10.1111/j.1528-1167.2010.02777.x 21219302 PMC3058866

[B13] Borges M. A. Min L. L. Guerreiro C. A. M. Yacubian E. M. T. Cordeiro J. A. Tognola W. A.. (2004). Urban prevalence of epilepsy: populational study in São José do Rio Preto, a medium-sized city in Brazil. Arq. Neuropsiquiatr. 62, 199–204. 10.1590/s0004-282x2004000200002 15235717

[B14] Bouilleret V. Ridoux V. Depaulis A. Marescaux C. Nehlig A. Le Gal La Salle G. (1999). Recurrent seizures and hippocampal sclerosis following intrahippocampal kainate injection in adult mice: electroencephalography, histopathology and synaptic reorganization similar to mesial temporal lobe epilepsy. Neuroscience 89, 717–729. 10.1016/s0306-4522(98)00401-1 10199607

[B15] Brodie M. J. Barry S. J. E. Bamagous G. A. Norrie J. D. Kwan P. (2012). Patterns of treatment response in newly diagnosed epilepsy. Neurology 78, 1548–1554. 10.1212/WNL.0b013e3182563b19 22573629 PMC3348850

[B16] Canevini M. P. Kotulska-Jozwiak K. Curatolo P. La Briola F. Peron A. Słowińska M.. (2018). Current concepts on epilepsy management in tuberous sclerosis complex. Am. J. Med. Genet. C Semin. Med. Genet. 178, 299–308. 10.1002/ajmg.c.31652 30255982

[B17] Canto A. M. Matos A. H. B. Godoi A. B. Vieira A. S. Aoyama B. B. Rocha C. S.. (2020). Multi-omics analysis suggests enhanced epileptogenesis in the Cornu Ammonis 3 of the pilocarpine model of mesial temporal lobe epilepsy. Hippocampus [Epub ahead of print]. 10.1002/hipo.23268 33037862

[B18] Cavalheiro E. A. Leite J. P. Bortolotto Z. A. Turski W. A. Ikonomidou C. Turski L. (1991). Long-term effects of pilocarpine in rats: structural damage of the brain triggers kindling and spontaneous recurrent seizures. Epilepsia 32, 778–782. 10.1111/j.1528-1157.1991.tb05533.x 1743148

[B19] Cendes F. (2005). Mesial temporal lobe epilepsy syndrome: an updated overview. J. Epilepsy Clin. Neurophysiol. 11, 141–144. 10.1586/ern.10.53 20518611

[B20] Chandana R. Mythri R. B. Mahadevan A. Shankar S. K. Srinivas Bharath M. M. (2009). Biochemical analysis of protein stability in human brain collected at different post-mortem intervals. Indian J. Med. Res. 129, 189–199. 19293447

[B21] Chapouthier G. Launay J.-M. Venault P. Breton C. Roubertoux P. L. Crusio W. E. (1998). Genetic selection of mouse lines differing in sensitivity to a benzodiazepine receptor inverse agonist. Brain Res. 787, 85–90. 10.1016/s0006-8993(97)01483-2 9518562

[B22] Che Y. Piao C. S. Han P.-L. Lee J.-K. (2001). Delayed induction of α B-crystallin in activated glia cells of hippocampus in kainic acid-treated mouse brain. J. Neurosci. Res. 65, 425–431. 10.1002/jnr.1170 11536326

[B23] Cho S. Volpe B. T. Bae Y. Hwang O. Choi H. J. Gal J.. (1999). Blockade of tetrahydrobiopterin synthesis protects neurons after transient forebrain ischemia in rat: a novel role for the cofactor. J. Neurosci. 19, 878–889. 10.1523/JNEUROSCI.19-03-00878.1999 9920651 PMC6782138

[B24] Crepeau A. Z. Sirven J. I. (2017). Management of adult onset seizures. Mayo Clin. Proc. 92, 306–318. 10.1016/j.mayocp.2016.11.013 28160877

[B25] Crespel A. Coubes P. Rousset M.-C. Brana C. Rougier A. Rondouin G.. (2002). Inflammatory reactions in human medial temporal lobe epilepsy with hippocampal sclerosis. Brain Res. 952, 159–169. 10.1016/s0006-8993(02)03050-0 12376176

[B26] Curatolo P. Nabbout R. Lagae L. Aronica E. Ferreira J. C. Feucht M.. (2018). Management of epilepsy associated with tuberous sclerosis complex: updated clinical recommendations. Eur. J. Paediatr. Neurol. 22, 738–748. 10.1016/j.ejpn.2018.05.006 29880258

[B27] Curia G. Longo D. Biagini G. Jones R. S. G. Avoli M. (2008). The pilocarpine model of temporal lobe epilepsy. J. Neurosci. Methods 172, 143–157. 10.1016/j.jneumeth.2008.04.019 18550176 PMC2518220

[B28] Czech T. Yang J.-W. Csaszar E. Kappler J. Baumgartner C. Lubec G. (2004). Reduction of hippocampal collapsin response mediated protein-2 in patients with mesial temporal lobe epilepsy. Neurochem. Res. 29, 2189–2196. 10.1007/s11064-004-7025-3 15672539

[B29] Danış Ö. Demir S. Günel A. Aker R. G. Gülçebi M. Onat F.. (2011). Changes in intracellular protein expression in cortex, thalamus and hippocampus in a genetic rat model of absence epilepsy. Brain Res. Bull. 84, 381–388. 10.1016/j.brainresbull.2011.02.002 21310218

[B30] Deeba F. Sanz-Leon P. Robinson P. A. (2018). Dependence of absence seizure dynamics on physiological parameter evolution. J. Theor. Biol. 454, 11–21. 10.1016/j.jtbi.2018.05.029 29807025

[B31] Depaulis A. van Luijtelaar G. (2006). “Genetic models of absence epilepsy in the rat,” in Models of Seizures and Epilepsy, eds Pitkänen A. Schwartkroin P. A. Moshé S. L. (Amsterdam: Elsevier), 233–248.

[B32] des Portes V. Francis F. Pinard J.-M. Desguerre I. Moutard M.-L. Snoeck I.. (1998). Doublecortin is the major gene causing X-linked subcortical laminar heterotopia (SCLH). Hum. Mol. Genet. 7, 1063–1070. 10.1093/hmg/7.7.1063 9618162

[B33] Devinsky O. Vezzani A. Najjar S. De Lanerolle N. C. Rogawski M. A. (2013). Glia and epilepsy: excitability and inflammation. Trends Neurosci. 36, 174–184. 10.1016/j.tins.2012.11.008 23298414

[B34] Devinsky O. Vezzani A. O’Brien T. J. Jette N. Scheffer I. E. de Curtis M.. (2018). Epilepsy. Nat. Rev. Dis. Primer 4:18024. 10.1038/nrdp.2018.24 29722352

[B35] do Canto A. M. Vieira A. S. Matos A. H. B. Carvalho B. S. Henning B. Norwood B. A.. (2020). Laser microdissection-based microproteomics of the hippocampus of a rat epilepsy model reveals regional differences in protein abundances. Sci. Rep. 10:4412. 10.1038/s41598-020-61401-8 32157145 PMC7064578

[B36] Dombkowski A. A. Batista C. E. Cukovic D. Carruthers N. J. Ranganathan R. Shukla U.. (2016). Cortical tubers: windows into dysregulation of epilepsy risk and synaptic signaling genes by MicroRNAs. Cereb. Cortex 26, 1059–1071. 10.1093/cercor/bhu276 25452577 PMC4737604

[B37] Eid T. Thomas M. Spencer D. Rundén-Pran E. Lai J. Malthankar G.. (2004). Loss of glutamine synthetase in the human epileptogenic hippocampus: possible mechanism for raised extracellular glutamate in mesial temporal lobe epilepsy. Lancet 363, 28–37. 10.1016/s0140-6736(03)15166-5 14723991

[B38] ElHajj Z. Cachot A. Müller T. Riederer I. M. Riederer B. M. (2016). Effects of postmortem delays on protein composition and oxidation. Brain Res. Bull. 121, 98–104. 10.1016/j.brainresbull.2016.01.005 26791740

[B39] England M. J. Liverman C. T. Schultz A. M. Strawbridge L. M. (2012). Epilepsy across the spectrum: promoting health and understanding. Epilepsy Behav. 25, 266–276. 10.1016/j.yebeh.2012.06.016 23041175 PMC3548323

[B40] Ferreira P. G. Muñoz-Aguirre M. Reverter F. Sá Godinho C. P. Sousa A. Amadoz A.. (2018). The effects of death and post-mortem cold ischemia on human tissue transcriptomes. Nat. Commun. 9:490. 10.1038/s41467-017-02772-x 29440659 PMC5811508

[B41] Fisher R. S. Acevedo C. Arzimanoglou A. Bogacz A. Cross J. H. Elger C. E.. (2014). ILAE official report: a practical clinical definition of epilepsy. Epilepsia 55, 475–482. 10.1111/epi.12550 24730690

[B42] Fukata Y. Fukata M. (2017). Epilepsy and synaptic proteins. Curr. Opin. Neurobiol. 45, 1–8. 10.1016/j.conb.2017.02.001 28219682

[B43] Furtinger S. Pirker S. Czech T. Baumgartner C. Ransmayr G. Sperk G. (2001). Plasticity of Y1 and Y2 receptors and neuropeptide Y fibers in patients with temporal lobe epilepsy. J. Neurosci. 21, 5804–5812. 10.1523/JNEUROSCI.21-15-05804.2001 11466452 PMC6762672

[B44] Gales J. M. Prayson R. A. (2017). Chronic inflammation in refractory hippocampal sclerosis-related temporal lobe epilepsy. Ann. Diagn. Pathol. 30, 12–16. 10.1016/j.anndiagpath.2017.05.009 28965622

[B45] Gano L. B. Liang L.-P. Ryan K. Michel C. R. Gomez J. Vassilopoulos A.. (2018). Altered mitochondrial acetylation profiles in a kainic acid model of temporal lobe epilepsy. Free Radic. Biol. Med. 123, 116–124. 10.1016/j.freeradbiomed.2018.05.063 29778462 PMC6082368

[B46] Garcia C. C. Blair H. J. Seager M. Coulthard A. Tennant S. Buddles M.. (2004). Identification of a mutation in synapsin I, a synaptic vesicle protein, in a family with epilepsy. J. Med. Genet. 41, 183–186. 10.1136/jmg.2003.013680 14985377 PMC1735688

[B47] Gatzonis S. D. Roupakiotis S. Kambayianni E. Politi A. Triantafyllou N. Mantouvalos V.. (2002). Hemispheric predominance of abnormal findings in electroencephalogram (EEG). Seizure 11, 442–444. 10.1053/seiz.2001.0642 12237070

[B48] Gorter J. A. Lopes da Silva F. H. (2002). “Abnormal plastic changes in a rat model for mesial temporal lobe epilepsy: a short review,” in Progress in Brain Research, eds Hofman M. A. Boer G. J. Holtmaat A. J. G. D. Van Someren E. J. W. Verhaagenand J. Swaab D. F. (Amsterdam, Netherlands: Elsevier), 61–72. 10.1016/s0079-6123(02)38071-3 12432763

[B49] Gøtzsche C. R. Nikitidou L. Sørensen A. T. Olesen M. V. Sørensen G. Christiansen S. H. O.. (2012). Combined gene overexpression of neuropeptide Y and its receptor Y5 in the hippocampus suppresses seizures. Neurobiol. Dis. 45, 288–296. 10.1016/j.nbd.2011.08.012 21884793

[B50] Greenberg D. A. Jin K. (2005). From angiogenesis to neuropathology. Nature 438, 954–959. 10.1038/nature04481 16355213

[B51] Greene N. D. E. Bamidele A. Choy M. de Castro S. C. P. Wait R. Leung K.-Y.. (2007). Proteome changes associated with hippocampal MRI abnormalities in the lithium pilocarpine-induced model of convulsive status epilepticus. Proteomics 7, 1336–1344. 10.1002/pmic.200601027 17366478

[B52] Grone B. P. Baraban S. C. (2015). Animal models in epilepsy research: legacies and new directions. Nat. Neurosci. 18, 339–343. 10.1038/nn.3934 25710835

[B53] Hagemann T. L. Boelens W. C. Wawrousek E. F. Messing A. (2009). Suppression of GFAP toxicity by αB-crystallin in mouse models of Alexander disease. Hum. Mol. Genet. 18, 1190–1199. 10.1093/hmg/ddp013 19129171 PMC2655774

[B54] Hauser W. A. Annegers J. F. Rocca W. A. (1996). Descriptive epidemiology of epilepsy: contributions of population-based studies from Rochester, Minnesota. Mayo Clin. Proc. 71, 576–586. 10.4065/71.6.576 8642887

[B55] He S. Wang Q. He J. Pu H. Yang W. Ji J. (2006). Proteomic analysis and comparison of the biopsy and autopsy specimen of human brain temporal lobe. Proteomics 6, 4987–4996. 10.1002/pmic.200600078 16912969

[B56] Heinrich C. Lähteinen S. Suzuki F. Anne-Marie L. Huber S. Häussler U.. (2011). Increase in BDNF-mediated TrkB signaling promotes epileptogenesis in a mouse model of mesial temporal lobe epilepsy. Neurobiol. Dis. 42, 35–47. 10.1016/j.nbd.2011.01.001 21220014

[B57] Higurashi M. Iketani M. Takei K. Yamashita N. Aoki R. Kawahara N.. (2012). Localized role of CRMP1 and CRMP2 in neurite outgrowth and growth cone steering. Dev. Neurobiol. 72, 1528–1540. 10.1002/dneu.22017 22378692

[B58] Hong E.-H. Kim J.-Y. Kim J.-H. Lim D.-S. Kim M. Kim J.-Y. (2018). BIG2-ARF1-RhoA-mDia1 signaling regulates dendritic golgi polarization in hippocampal neurons. Mol. Neurobiol. 55, 7701–7716. 10.1007/s12035-018-0954-7 29455446

[B59] Hosp F. Mann M. (2017). A primer on concepts and applications of proteomics in neuroscience. Neuron 96, 558–571. 10.1016/j.neuron.2017.09.025 29096073

[B60] Hsu D. (2007). “The dentate gyrus as a filter or gate: a look back and a look ahead,” in Progress in Brain Research, ed Scharfman H. E. (Amsterdam, Netherlands: Elsevier), 601–613. 10.1016/S0079-6123(07)63032-5 17765740

[B61] Isokawa M. Levesque M. Babb T. Engel J. (1993). Single mossy fiber axonal systems of human dentate granule cells studied in hippocampal slices from patients with temporal lobe epilepsy. J. Neurosci. 13, 1511–1522. 10.1523/JNEUROSCI.13-04-01511.1993 8463831 PMC6576742

[B62] Jaber Z. Aouad P. Al Medawar M. Bahmad H. Abou-Abbass H. Ghandour H. (2016a). “Role of systems biology in brain injury biomarker discovery: neuroproteomics application,” in Injury Models of the Central Nervous System Methods in Molecular Biology, eds Kobeissy F. H. Dixon C. E. Hayes R. L. Mondello S. (New York, NY: Springer), 157–174. 10.1007/978-1-4939-3816-2_10 27604718

[B63] Jaber Z. Aouad P. Al Medawar M. Bahmad H. Abou-Abbass H. Kobeissy F. (2016b). “Application of systems biology to neuroproteomics: the path to enhanced theranostics in traumatic brain injury,” in Injury Models of the Central Nervous System Methods in Molecular Biology, eds Kobeissy F. H. Dixon C. E. Hayes R. L. Mondello S. (New York, NY: Springer), 139–155. 10.1007/978-1-4939-3816-2_9 27604717

[B64] Jan L. Y. Jan Y. N. (1997). Cloned potassium channels from eukaryotes and prokaryotes. Annu. Rev. Neurosci. 20, 91–123. 10.1146/annurev.neuro.20.1.91 9056709

[B65] Junker H. Späte K. Suofu Y. Walther R. Schwarz G. Kammer W.. (2005). Proteomic identification of the involvement of the mitochondrial rieske protein in epilepsy. Epilepsia 46, 339–343. 10.1111/j.0013-9580.2005.46904.x 15730530

[B66] Kalwy S. A. Akbar M. T. Coffin R. S. de Belleroche J. Latchman D. S. (2003). Heat shock protein 27 delivered *via* a herpes simplex virus vector can protect neurons of the hippocampus against kainic-acid-induced cell loss. Mol. Brain Res. 111, 91–103. 10.1016/s0169-328x(02)00692-7 12654509

[B67] Keck M. van Dijk R. M. Deeg C. A. Kistler K. Walker A. von Rüden E.-L.. (2018). Proteomic profiling of epileptogenesis in a rat model: focus on cell stress, extracellular matrix and angiogenesis. Neurobiol. Dis. 112, 119–135. 10.1016/j.nbd.2018.01.013 29413716

[B68] Keren-Aviram G. Dachet F. Bagla S. Balan K. Loeb J. A. Dratz E. A. (2018). Proteomic analysis of human epileptic neocortex predicts vascular and glial changes in epileptic regions. PLoS One 13:e0195639. 10.1371/journal.pone.0195639 29634780 PMC5892923

[B69] Kilb W. Kirischuk S. Luhmann H. J. (2011). Electrical activity patterns and the functional maturation of the neocortex: shaping of developing cortical circuits by electrical activity. Eur. J. Neurosci. 34, 1677–1686. 10.1111/j.1460-9568.2011.07878.x 22103424

[B70] Kim S. I. Voshol H. van Oostrum J. Hastings T. G. Cascio M. Glucksman M. J. (2004). Neuroproteomics: expression profiling of the brain’s proteomes in health and disease. Neurochem. Res. 29, 1317–1331. 10.1023/b:nere.0000023618.35579.7c 15176488

[B71] Kokaia M. (2011). Seizure-induced neurogenesis in the adult brain: seizure-induced neurogenesis. Eur. J. Neurosci. 33, 1133–1138. 10.1111/j.1460-9568.2011.07612.x 21395857

[B72] Kovac S. Abramov A. Y. Walker M. C. (2013). Energy depletion in seizures: anaplerosis as a strategy for future therapies. Neuropharmacology 69, 96–104. 10.1016/j.neuropharm.2012.05.012 22659085

[B73] Kruman I. I. Schwartz E. Kruman Y. Cutler R. G. Zhu X. Greig N. H.. (2004). Suppression of uracil-DNA glycosylase induces neuronal apoptosis. J. Biol. Chem. 279, 43952–43960. 10.1074/jbc.M408025200 15297456

[B74] Kumar G. K. Prabhakar N. R. (2008). Post-translational modification of proteins during intermittent hypoxia. Respir. Physiol. Neurobiol. 164, 272–276. 10.1016/j.resp.2008.05.017 18602876 PMC2642904

[B75] Kunz W. S. Kudin A. P. Vielhaber S. Blümcke I. Zuschratter W. Schramm J.. (2000). Mitochondrial complex I deficiency in the epileptic focus of patients with temporal lobe epilepsy. Ann. Neurol. 48, 766–773. 10.1002/1531-8249(200011)48:5<766::aid-ana10>3.0.co;2-m 11079540

[B76] Kwan P. Arzimanoglou A. Berg A. T. Brodie M. J. Allen Hauser W. Mathern G.. (2010). Definition of drug resistant epilepsy: consensus proposal by the *ad hoc* Task Force of the ILAE Commission on Therapeutic Strategies. Epilepsia 51, 1069–1077. 10.1111/j.1528-1167.2009.02397.x 19889013

[B77] Lagarrigue M. Alexandrov T. Dieuset G. Perrin A. Lavigne R. Baulac S.. (2012). New analysis workflow for MALDI imaging mass spectrometry: application to the discovery and identification of potential markers of childhood absence epilepsy. J. Proteome Res. 11, 5453–5463. 10.1021/pr3006974 22994238

[B78] Laidlaw J. (ed.). (1988). A Textbook of Epidemiology. 3rd Edn. Edinburgh: Churchill Livingstone.

[B79] Leal R. B. Lopes M. W. Formolo D. A. de Carvalho C. R. Hoeller A. A. Latini A.. (2020). Amygdala levels of the GluA1 subunit of glutamate receptors and its phosphorylation state at serine 845 in the anterior hippocampus are biomarkers of ictal fear but not anxiety. Mol. Psychiatry 25, 655–665. 10.1038/s41380-018-0084-7 29880883

[B80] Lee A. Maldonado M. Baybis M. Walsh C. A. Scheithauer B. Yeung R.. (2003). Markers of cellular proliferation are expressed in cortical tubers. Ann. Neurol. 53, 668–673. 10.1002/ana.10579 12731003

[B81] Leite J. P. Chimelli L. Terra-Bustamante V. C. Costa E. T. Assirati J. A. De Nucci G.. (2002). Loss and sprouting of nitric oxide synthase neurons in the human epileptic hippocampus. Epilepsia 43, 235–242. 10.1046/j.1528-1157.43.s.5.29.x 12121328

[B82] Lévesque M. Shiri Z. Chen L.-Y. Avoli M. (2018). High-frequency oscillations and mesial temporal lobe epilepsy. Neurosci. Lett. 667, 66–74. 10.1016/j.neulet.2017.01.047 28115239

[B83] Li A. Choi Y.-S. Dziema H. Cao R. Cho H.-Y. Jung Y. J.. (2010). Proteomic profiling of the epileptic dentate gyrus. Brain Pathol. Zurich Switz. 20, 1077–1089. 10.1111/j.1750-3639.2010.00414.x 20608933 PMC2951482

[B84] Li K. W. Ganz A. B. Smit A. B. (2019). Proteomics of neurodegenerative diseases: analysis of human post-mortem brain. J. Neurochem. 151, 435–445. 10.1111/jnc.14603 30289976 PMC6899881

[B86] Liu Y.-D. Ma M.-Y. Hu X.-B. Yan H. Zhang Y.-K. Yang H.-X.. (2020). Brain proteomic profiling in intractable epilepsy caused by TSC1 truncating mutations: a small sample study. Front. Neurol. 11:475. 10.3389/fneur.2020.00475 32655475 PMC7326032

[B85] Liu X.-Y. Yang J.-L. Chen L.-J. Zhang Y. Yang M.-L. Wu Y.-Y.. (2008). Comparative proteomics and correlated signaling network of rat hippocampus in the pilocarpine model of temporal lobe epilepsy. Proteomics 8, 582–603. 10.1002/pmic.200700514 18186018

[B87] Löscher W. (1984). Genetic animal models of epilepsy as a unique resource for the evaluation of anticonvulsant drugs. A review. Methods Find. Exp. Clin. Pharmacol. 6, 531–547. 10.1016/j.chemosphere.2020.129167 6439966

[B88] Löscher W. (2011). Critical review of current animal models of seizures and epilepsy used in the discovery and development of new antiepileptic drugs. Seizure 20, 359–368. 10.1016/j.seizure.2011.01.003 21292505

[B89] Löscher W. (2017). Animal models of seizures and epilepsy: past, present, and future role for the discovery of antiseizure drugs. Neurochem. Res. 42, 1873–1888. 10.1007/s11064-017-2222-z 28290134

[B90] Löscher W. Schmidt D. (2011). Modern antiepileptic drug development has failed to deliver: ways out of the current dilemma: ways out of the current dilemma with new AEDs. Epilepsia 52, 657–678. 10.1111/j.1528-1167.2011.03024.x 21426333

[B91] Luan L. Sun Y. Yang K. (2018). Surgical strategy for temporal lobe epilepsy with dual pathology and incomplete evidence from EEG and neuroimaging. Exp. Ther. Med. 16, 4886–4892. 10.3892/etm.2018.6774 30546403 PMC6256846

[B92] Luhmann H. J. Kilb W. Clusmann H. (2014). “Malformations of cortical development and neocortical focus,” in International Review of Neurobiology, eds Premysl Jiruska Marco de Curtis Jefferys J. G. R. (San Diego, CA: Elsevier), 35–61. 10.1016/B978-0-12-418693-4.00003-0 25078498

[B93] Magalhães P. H. M. Moraes H. T. Athie M. C. P. Secolin R. Lopes-Cendes I. (2019). New avenues in molecular genetics for the diagnosis and application of therapeutics to the epilepsies. Epilepsy Behav. [Epub ahead of print]. 10.1016/j.yebeh.2019.07.029 31400936

[B94] Maksimenko V. A. van Heukelum S. Makarov V. V. Kelderhuis J. Lüttjohann A. Koronovskii A. A.. (2017). Absence seizure control by a brain computer interface. Sci. Rep. 7:2487. 10.1038/s41598-017-02626-y 28555070 PMC5447660

[B95] Mériaux C. Franck J. Park D. B. Quanico J. Kim Y. H. Chung C. K.. (2014). Human temporal lobe epilepsy analyses by tissue proteomics. Hippocampus 24, 628–642. 10.1002/hipo.22246 24449190

[B96] Mota M. V. B. Zaidan B. C. do Canto A. M. Ghizoni E. Tedeschi H. de Souza Queiroz L.. (2019). ATP synthase subunit β immunostaining is reduced in the sclerotic hippocampus of epilepsy patients. Cell. Mol. Neurobiol. 39, 149–160. 10.1007/s10571-018-0641-2 30539418 PMC11469864

[B97] Nabeka H. Uematsu K. Takechi H. Shimokawa T. Yamamiya K. Li C.. (2014). Prosaposin overexpression following kainic acid-induced neurotoxicity. PLoS One 9:e110534. 10.1371/journal.pone.0110534 25461957 PMC4251898

[B98] Nagy C. Maheu M. Lopez J. P. Vaillancourt K. Cruceanu C. Gross J. A.. (2015). Effects of postmortem interval on biomolecule integrity in the brain. J. Neuropathol. Exp. Neurol. 74, 459–469. 10.1097/NEN.0000000000000190 25868148

[B99] Niere F. Namjoshi S. Song E. Dilly G. A. Schoenhard G. Zemelman B. V.. (2016). Analysis of proteins that rapidly change upon mechanistic/mammalian target of rapamycin complex 1 (mTORC1) repression identifies Parkinson protein 7 (PARK7) as a novel protein aberrantly expressed in tuberous sclerosis complex (TSC). Mol. Cell. Proteomics 15, 426–444. 10.1074/mcp.M115.055079 26419955 PMC4739665

[B100] Norwood B. A. Bumanglag A. V. Osculati F. Sbarbati A. Marzola P. Nicolato E.. (2010). Classic hippocampal sclerosis and hippocampal-onset epilepsy produced by a single “cryptic” episode of focal hippocampal excitation in awake rats. J. Comp. Neurol. 518, 3381–3407. 10.1002/cne.22406 20575073 PMC2894278

[B101] Oberheim N. A. Tian G.-F. Han X. Peng W. Takano T. Ransom B.. (2008). Loss of astrocytic domain organization in the epileptic brain. J. Neurosci. 28, 3264–3276. 10.1523/JNEUROSCI.4980-07.2008 18367594 PMC6670598

[B102] Ottman R. Winawer M. R. Kalachikov S. Barker-Cummings C. Gilliam T. C. Pedley T. A.. (2004). LGI1 mutations in autosomal dominant partial epilepsy with auditory features. Neurology 62, 1120–1126. 10.1212/01.wnl.0000120098.39231.6e 15079011 PMC1361770

[B103] Ousman S. S. Tomooka B. H. van Noort J. M. Wawrousek E. F. O’Conner K. Hafler D. A.. (2007). Protective and therapeutic role for αB-crystallin in autoimmune demyelination. Nature 448, 474–479. 10.1038/nature05935 17568699

[B104] Palmini A. Andermann F. Tampieri D. Andermann E. Robitaille Y. Olivier A. (1992). Epilepsy and cortical cytoarchitectonic abnormalities: an attempt at correlating basic mechanisms with anatomoclinical syndromes. Epilepsy Res. Suppl. 9, 19–29; discussion 29–30. 1337438

[B105] Papageorgiou I. E. Gabriel S. Fetani A. F. Kann O. Heinemann U. (2011). Redistribution of astrocytic glutamine synthetase in the hippocampus of chronic epileptic rats. Glia 59, 1706–1718. 10.1002/glia.21217 21780187

[B106] Perez-Olle R. Lopez-Toledano M. A. Liem R. K. H. (2004). The G336S variant in the human neurofilament-M gene does not affect its assembly or distribution: importance of the functional analysis of neurofilament variants. J. Neuropathol. Exp. Neurol. 63, 759–774. 10.1093/jnen/63.7.759 15290901

[B107] Persike D. Lima M. Amorim R. Cavalheiro E. Yacubian E. Centeno R. (2012). Hippocampal proteomic profile in temporal lobe epilepsy. J. Epilepsy Clin. Neurophysiol. 18, 53–56. 10.1590/s1676-26492012000200007

[B108] Qin L. Liu X. Liu S. Liu Y. Yang Y. Yang H.. (2017). Differentially expressed proteins underlying childhood cortical dysplasia with epilepsy identified by iTRAQ proteomic profiling. PLoS One 12:e0172214. 10.1371/journal.pone.0172214 28222113 PMC5319751

[B109] Ramadan N. Ghazale H. El-Sayyad M. El-Haress M. Kobeissy F. H. (2017). “Neuroproteomics studies: challenges and updates,” in Neuroproteomics Methods in Molecular Biology, eds Kobeissy F. H. Stevens S. M. (New York, NY: Springer), 3–19. 10.1007/978-1-4939-6952-4_1 28508355

[B110] Ramanjaneyulu R. Ticku M. K. (1984). Interactions of pentamethylenetetrazole and tetrazole analogues with the picrotoxinin site of the benzodiazepine-gaba receptor-ionophore complex. Eur. J. Pharmacol. 98, 337–345. 10.1016/0014-2999(84)90282-6 6327331

[B111] Rose G. Dato S. Altomare K. Bellizzi D. Garasto S. Greco V.. (2003). Variability of the SIRT3 gene, human silent information regulator Sir2 homologue, and survivorship in the elderly. Exp. Gerontol. 38, 1065–1070. 10.1016/s0531-5565(03)00209-2 14580859

[B112] Rowley S. Patel M. (2013). Mitochondrial involvement and oxidative stress in temporal lobe epilepsy. Free Radic. Biol. Med. 62, 121–131. 10.1016/j.freeradbiomed.2013.02.002 23411150 PMC4043127

[B113] Sadeghi L. Rizvanov A. A. Salafutdinov I. I. Dabirmanesh B. Sayyah M. Fathollahi Y.. (2017). Hippocampal asymmetry: differences in the left and right hippocampus proteome in the rat model of temporal lobe epilepsy. J. Proteomics 154, 22–29. 10.1016/j.jprot.2016.11.023 27932302

[B114] Sánchez R. G. Parrish R. R. Rich M. Webb W. M. Lockhart R. M. Nakao K.. (2019). Human and rodent temporal lobe epilepsy is characterized by changes in O-GLCNAC homeostasis that can be reversed to dampen epileptiform activity. Neurosci. Dis. 124, 531–543. 10.1016/j.nbd.2019.01.001 30625365 PMC6379093

[B115] Sarnat H. B. Flores-Sarnat L. (2009). α-B-crystallin as a tissue marker of epileptic foci in paediatric resections. Can. J. Neurol. Sci. 36, 566–574. 10.1017/s0317167100008052 19831124

[B116] Saxena A. Sampson J. (2015). Epilepsy in tuberous sclerosis: phenotypes, mechanisms, and treatments. Semin. Neurol. 35, 269–276. 10.1055/s-0035-1552616 26060906

[B117] Schwartzkroin P. A. Wenzel H. J. (2012). Are developmental dysplastic lesions epileptogenic? Are dysplasias epileptogenic? Epilepsia 53, 35–44. 10.1111/j.1528-1167.2012.03473.x 22612807

[B118] Sharma A. K. Reams R. Y. Jordan W. H. Miller M. A. Thacker H. L. Snyder P. W. (2007). Mesial temporal lobe epilepsy: pathogenesis, induced rodent models and lesions. Toxicol. Pathol. 35, 984–999. 10.1080/01926230701748305 18098044

[B119] Shepherd C. Liu J. Goc J. Martinian L. Jacques T. S. Sisodiya S. M.. (2013). A quantitative study of white matter hypomyelination and oligodendroglial maturation in focal cortical dysplasia type II. Epilepsia 54, 898–908. 10.1111/epi.12143 23551043 PMC4165267

[B120] Singh A. Trevick S. (2016). The epidemiology of global epilepsy. Neurol. Clin. 34, 837–847. 10.1016/j.ncl.2016.06.015 27719996

[B121] Sousa J. S. D’Imprima E. Vonck J. (2018). “Mitochondrial respiratory chain complexes,” in Membrane Protein Complexes: Structure and Function, Subcellular Biochemistry, eds Harris J. R. Boekema E. J. (Singapore: Springer Singapore), 167–227. 10.1007/978-981-10-7757-9_7 29464561

[B122] Spiliotis E. T. (2006). Here come the septins: novel polymers that coordinate intracellular functions and organization. J. Cell Sci. 119, 4–10. 10.1242/jcs.02746 16371649 PMC3368708

[B123] Staley K. (2015). Molecular mechanisms of epilepsy. Nat. Neurosci. 18, 367–372. 10.1038/nn.3947 25710839 PMC4409128

[B124] Takechi K. Suemaru K. Kawasaki H. Araki H. (2012). Impaired memory following repeated pentylenetetrazol treatments in kindled mice. Yakugaku Zasshi 132, 179–182. 10.1248/yakushi.132.179 22293696

[B125] Tassi L. Meroni A. Deleo F. Villani F. Mai R. Lo Russo G.. (2009). Temporal lobe epilepsy: neuropathological and clinical correlations in 243 surgically treated patients. Epileptic. Disord. 11, 281–292. 10.1684/epd.2009.0279 19945931

[B126] Thijs R. D. Surges R. O’Brien T. J. Sander J. W. (2019). Epilepsy in adults. Lancet 393, 689–701. 10.1016/S0140-6736(18)32596-0 30686584

[B127] Tokuraku K. Noguchi T. Q. P. Nishie M. Matsushima K. Kotani S. (2007). An isoform of microtubule-associated protein 4 inhibits kinesin-driven microtubule gliding. J. Biochem. 141, 585–591. 10.1093/jb/mvm063 17317690

[B128] Visanji N. P. Wong J. C. Wang S. X. Cappel B. Kleinschmidt-DeMasters B. K. Handler M. H.. (2012). A proteomic analysis of pediatric seizure cases associated with astrocytic inclusions: Proteomics Seizure Astrocytic Inclusions. Epilepsia 53, e50–e54. 10.1111/j.1528-1167.2011.03369.x 22220588

[B129] Walker A. Russmann V. Deeg C. A. von Toerne C. Kleinwort K. J. H. Szober C.. (2016). Proteomic profiling of epileptogenesis in a rat model: focus on inflammation. Brain. Behav. Immun. 53, 138–158. 10.1016/j.bbi.2015.12.007 26685804

[B131] Wang X. Phelan S. A. Forsman-Semb K. Taylor E. F. Petros C. Brown A.. (2003). Mice with targeted mutation of peroxiredoxin 6 develop normally but are susceptible to oxidative stress. J. Biol. Chem. 278, 25179–25190. 10.1074/jbc.M302706200 12732627

[B130] Wang L. Song L.-F. Chen X.-Y. Ma Y.-L. Suo J.-F. Shi J.-H.. (2019). MiR-181b inhibits P38/JNK signaling pathway to attenuate autophagy and apoptosis in juvenile rats with kainic acid-induced epilepsy *via* targeting TLR4. CNS Neurosci. Ther. 25, 112–122. 10.1111/cns.12991 29808547 PMC6436603

[B132] Will J. L. Eckart M. T. Rosenow F. Bauer S. Oertel W. H. Schwarting R. K. W.. (2013). Enhanced sequential reaction time task performance in a rat model of mesial temporal lobe epilepsy with classic hippocampal sclerosis. Behav. Brain Res. 247, 65–72. 10.1016/j.bbr.2013.03.019 23511251

[B133] Wilson S. M. Khanna R. (2015). Specific binding of lacosamide to collapsin response mediator protein 2 (CRMP2) and direct impairment of its canonical function: implications for the therapeutic potential of lacosamide. Mol. Neurobiol. 51, 599–609. 10.1007/s12035-014-8775-9 24944082 PMC4272652

[B134] World Federation of Neurology World Health Organization (2017). Atlas: Country Resources for Neurological Disorders. 2nd Edn. Geneva: World Health Organization.

[B135] Wu Q. Liu J. Fang A. Li R. Bai Y. Kriegstein A. R.. (2014). The dynamics of neuronal migration. Adv. Exp. Med. Biol. 800, 25–36. 10.1007/978-94-007-7687-6_2 24243098

[B136] Xi J. Bai F. McGaha R. Andley U. P. (2006). α-crystallin expression affects microtubule assembly and prevents their aggregation. FASEB J. 20, 846–857. 10.1096/fj.05-5532com 16675842

[B137] Yamada J. Kurata A. Hirata M. Taniguchi T. Takama H. Furihata T.. (1999). Purification, molecular cloning, and genomic organization of human brain long-chain Acyl-CoA hydrolase. J. Biochem. 126, 1013–1019. 10.1093/oxfordjournals.jbchem.a022544 10578051

[B138] Yamashita N. Uchida Y. Ohshima T. Hirai S. Nakamura F. Taniguchi M.. (2006). Collapsin response mediator protein 1 mediates reelin signaling in cortical neuronal migration. J. Neurosci. 26, 13357–13362. 10.1523/JNEUROSCI.4276-06.2006 17182786 PMC6674993

[B139] Yang J. W. Czech T. Felizardo M. Baumgartner C. Lubec G. (2006). Aberrant expression of cytoskeleton proteins in hippocampus from patients with mesial temporal lobe epilepsy. Amino Acids 30, 477–493. 10.1007/s00726-005-0281-y 16583313

[B140] Yang J. W. Czech T. Yamada J. Csaszar E. Baumgartner C. Slavc I.. (2004). Aberrant cytosolic acyl-CoA thioester hydrolase in hippocampus of patients with mesial temporal lobe epilepsy. Amino Acids 27, 269–275. 10.1007/s00726-004-0138-9 15592755

[B141] Yang J.-W. Czech T. Gelpi E. Lubec G. (2005). Extravasation of plasma proteins can confound interpretation of proteomic studies of brain: a lesson from apo A-I in mesial temporal lobe epilepsy. Mol. Brain Res. 139, 348–356. 10.1016/j.molbrainres.2005.06.010 16095751

[B142] Ye H. Kaszuba S. (2017). Inhibitory or excitatory? Optogenetic interrogation of the functional roles of GABAergic interneurons in epileptogenesis. J. Biomed. Sci. 24:93. 10.1186/s12929-017-0399-8 29202749 PMC5715558

[B143] Yokota H. (2019). Applications of proteomics in pharmaceutical research and development. Biochim. Biophys. Acta Proteins Proteom. 1867, 17–21. 10.1016/j.bbapap.2018.05.008 29753086

[B144] Yuce-Dursun B. Danis O. Demir S. Ogan A. Onat F. (2014). Proteomic changes in the cortex membrane fraction of genetic absence epilepsy rats from Strasbourg. J. Integr. Neurosci. 13, 633–644. 10.1142/S021963521450023X 25352154

[B145] Zhang Z. Wu S. Stenoien D. L. Paša-Tolić L. (2014). High-throughput proteomics. Annu. Rev. Anal. Chem. 7, 427–454. 10.1146/annurev-anchem-071213-020216 25014346

